# The In Vitro Antioxidant and Anti-Inflammatory Activities of Selected Australian Seagrasses

**DOI:** 10.3390/life14060710

**Published:** 2024-05-30

**Authors:** Matthew J. Perry, Mara Curic, Abigail L. Scott, Edita Ritmejerytė, Dyah U. C. Rahayu, Paul A. Keller, Michael Oelgemöller, Karma Yeshi, Phurpa Wangchuk

**Affiliations:** 1 College of Public Health, Medical and Veterinary Sciences, James Cook University, Cairns, QLD 4878, Australiakarma.yeshi@my.jcu.edu.au (K.Y.); 2 Australian Institute of Tropical Health and Medicine, James Cook University, Cairns, QLD 4878, Australia; 3 Hochschule Fresenius, Faculty of Chemistry & Biology, University of Applied Sciences, Limburger Strasse 2, 65510 Idstein, Germany; 4 Centre of Tropical Water & Aquatic Ecosystem Research, James Cook University, Cairns, QLD 4878, Australia; abbi.scott1@jcu.edu.au; 5 School of Chemistry and Molecular Bioscience, Molecular Horizons, University of Wollongong, Wollongong, NSW 2522, Australia

**Keywords:** *Zostera muelleri*, seagrass, antioxidant activity, anti-inflammatory activity, PBMC assay, natural product isolation, luteolin, apigenin

## Abstract

Recent studies have shown that seagrasses could possess potential applications in the treatment of inflammatory disorders. Five seagrass species (*Zostera muelleri*, *Halodule uninervis*, *Cymodocea rotundata*, *Syringodium isoetifolium*, and *Thalassia hemprichii*) from the Great Barrier Reef (QLD, Australia) were thus collected, and their preliminary antioxidant and anti-inflammatory activities were evaluated. From the acetone extracts of five seagrass species subjected to 1,1-diphenyl-2-picrylhydrazyl (DPPH) radical scavenging antioxidant assay, the extract of *Z. muelleri* had the highest activity (half minimal concentration of inhibition (IC_50_) = 138 µg/mL), with the aerial parts (IC_50_ = 119 µg/mL) possessing significantly higher antioxidant activity than the roots (IC_50_ ≥ 500 µg/mL). A human peripheral blood mononuclear cells (PBMCs) assay with bacterial lipopolysaccharide (LPS) activation and LEGENDplex cytokine analysis showed that the aerial extract of *Z. muelleri* significantly reduced the levels of inflammatory cytokines tumour necrosis factor alpha (TNF-α), interleukin (IL)-1β, and IL-6 by 29%, 74%, and 90%, respectively, relative to the LPS treatment group. The aerial extract was thus fractionated with methanol (MeOH) and hexane fraction, and purification of the MeOH fraction by HPLC led to the isolation of 4-hydroxybenzoic acid (**1**), luteolin (**2**), and apigenin (**3**) as its major constituents. These compounds have been previously shown to reduce levels of TNF-α, IL-1β, and IL-6 and represent some of the major bioactive components of *Z. muelleri* aerial parts. This investigation represents the first study of the antioxidant and anti-inflammatory properties of *Z. muelleri* and the first isolation of small molecules from this species. These results highlight the potential for using seagrasses in treating inflammation and the need for further investigation.

## 1. Introduction

Inflammation describes the natural healing process after the body sustains damage or is exposed to toxins or infection. This process is initiated by the body’s production of key inflammatory cytokines such as tumour necrosis factor alpha (TNF-α), interleukin (IL)-1β, and IL-6, which promote key inflammatory responses including vasodilation, immune cell recruitment, and sensitivity to pain [1]. Under normal conditions, inflammation is acute in nature, rapidly clearing the infection or healing damaged tissues, and resolves within a few days. In chronic inflammation, however, the response continues for months or years, causing serious damage to the body [2]. In addition to a reduction in quality of life experienced by people with rheumatoid arthritis, inflammatory bowel disease (IBD), and acne, more life-threatening chronic inflammatory disorders such as cancer, stroke, diabetes mellitus, kidney disease, and ischemic heart disease have accounted for over 50% of deaths globally in the past few decades [3].

Given the severity and importance of chronic inflammation, there is a continual search for more effective treatments. The most common treatments for inflammatory conditions in modern medicine are glucocorticoids (steroids) and non-steroidal anti-inflammatory drugs (NSAIDs) [4], which are typically taken as oral or topical treatments depending upon the site of inflammation. In addition, dietary changes are also important interventions due to the link between the modern diet and inflammation [5,6].

It is common knowledge that fruits, vegetables, and other plants are a rich source of vitamins, minerals, and bioactive secondary metabolites (metabolic products that are not necessary for growth, development, and reproduction, but are essential for ecological and other interactions), many of which have anti-inflammatory properties [7,8]. In addition to commonly consumed plant materials, phytochemical analyses of many seagrasses including *Halophila stipulacea* [9], *Thalassodendron ciliatum* [9], *Halodule uninervis* [9], *Halodule pinifolia* [10,11], *Halophila ovalis* [10], *Syringodium isoetifolium* [10,11], *Thalassia hemprichii* [11,12], *Cymodocea rotundata* [11], *Enhalus acoroides* [11], *Cymodocea serrulate* [11], and *Zostera muelleri* [13,14] have indicated seagrasses are also natural reservoirs of phenolics, flavonoids, sterols, alkaloids, saponins, tannins, terpenoids, and other bioactive secondary metabolites.

Seagrasses, like other plants, produce this wide variety of secondary metabolites as active principles and signalling molecules, especially polyphenols, to cope with various ecological stresses such as poor nutrient availability, ionising UV radiation, temperature, salinity, and to prevent predation and microbial infection [15,16]. Such secondary metabolites account for the diverse biological activities observed in seagrass extracts including antimicrobial, anti-inflammatory, antioxidant, antiviral, anticancer, anti-ageing, and hepatoprotective properties [17]. These bioactive properties of seagrasses have been utilised in traditional medicine systems in Africa [18,19], Indonesia [20,21], and India [22] for the treatment of many ailments such as itchiness, muscle pains, wounds, gastrointestinal complaints, heart and kidney disease, cancer, and as a mosquito repellent. These bioactivities and traditional medicinal uses highlight the potential for seagrasses in lead compound discovery and for the development of new products for the treatment of common diseases.

Seagrasses typically grow in shallow coastal waters, and it has been reported that seagrass meadows are dominant vegetation types along the Great Barrier Reef (GBR), Australia [23]. Given that Australia’s coastline in its tropical regions is within the most biodiverse region for seagrasses globally [24] and given the current lack of research on seagrasses and their bioactivities, this study aimed to collect several seagrass species from the GBR in Far-North Queensland (FNQ), perform preliminary antioxidant and anti-inflammatory tests, and isolate major secondary metabolites from the most bioactive species.

## 2. Materials and Methods

### 2.1. Materials

#### 2.1.1. Plant Materials

**Figure 1:**
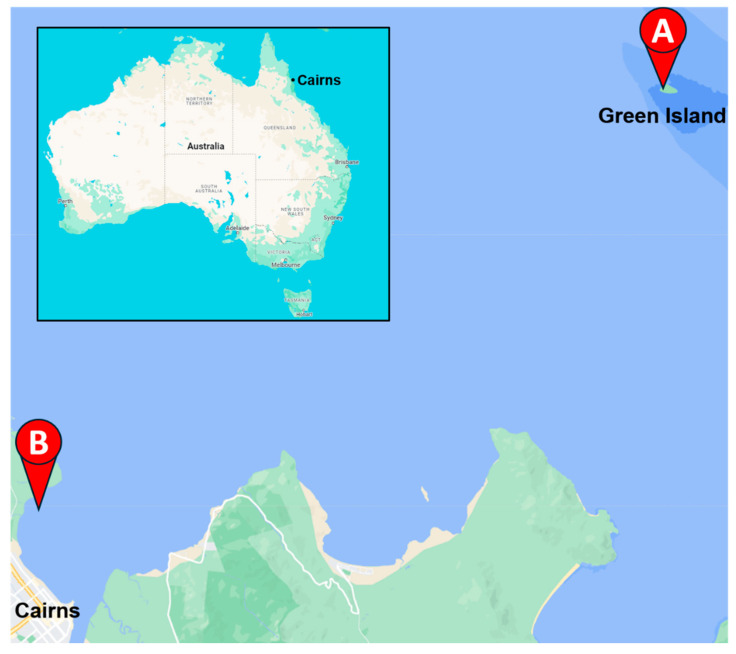
A map of seagrass collection locations around Cairns. The aerial and root parts of *Halodule uninervis*, *Cymodocea rotundata*, *Syringodium isoetifolium*, and *Thalassia hemprichii* were collected from Green Island (**A**), and *Zostera muelleri* was collected from Cairns harbour (**B**). Inset shows the location of Cairns in far north Queensland (FNQ), Australia. This image was created with the use of a map adapted from Google Maps.

The aerial parts and roots of *Thalassia hemprichii* (Ehrenb.) Asch., *Cymodocea rotundata* Asch. and Schweinf., *Halodule uninervis* (Forssk.) Boiss., and *Syringodium isoetifolium* (Asch.) Dandy were collected by Dr. Tim Smith and the TropWATER team on Green Island (16°45′31.1″ S, 145°58′21.8″ E) (Figure 1A) on 21 May 2021. The aerial components and roots of *Zostera muelleri* Irmisch ex Asch. were collected by Dr. Abbi Scott and the TropWATER team from Cairns Harbour, Queensland, Australia (16°53′25.5″ S, 145°46′06.1″ E) (Figure 1B) on 19 September 2023. All species were identified by Dr. Abbi Scott. The aerial parts and roots of all seagrass samples were washed, snap frozen, and stored in −80 °C until freeze-drying. Freeze-dried material was homogenised into a fine powder.

#### 2.1.2. Solvents, Reagents and Consumables

Methanol (MeOH) (ChemSupply Australia, Adelaide, Australia), acetone (ChemSupply Australia), trifluoroacetic acid (TFA) (Sigma-Aldrich, Lyon, France), and hexane fraction (hexanes) (ChemSupply Australia)were reagent grade and used without further purification (ChemSupply Australia, Adelaide, Australia). High-performance liquid chromatography (HPLC)-grade acetonitrile (MeCN) (ChemSupply Australia) was utilised for HPLC buffers. Liquid chromatography (LC)-grade MeOH (Labsolv, Samutsakorn, Thailand) was utilised for sample analysis by mass spectrometry (MS). Water used in HPLC buffers was purified by reverse osmosis (RO) (Sartorius Arium Comfort I UV, Göttingen, Germany).

The deuterated methanol (CD_3_OD) used for nuclear magnetic resonance (NMR) analysis was obtained from NovaChem (Cambridge Isotope Laboratories, Tewksbury, MA, USA).

The 1,1-diphenyl-2-picrylhydrazyl (DPPH) and gallic acid utilised in the antioxidant assays were obtained from Alfa Aesar (Ward Hill, Haverhill, MA, USA).

The following materials were used in the cell viability and anti-inflammatory assays: Ficoll-Paque PLUS density gradient (Stemcell Technologies, Vancouver, BC, Canada); heat-inactivated foetal bovine serum (FBS) (Corning, Melbourne, Australia) containing 10% dimethyl sulfoxide (DMSO) (Sigma Aldrich); RPMI-1640 (Gibco, Life Technologies, Burlington, ON, Canada); 100 U/mL penicillin (Gibco, Life Technologies); 100 μg/mL streptomycin (Gibco, Life Technologies); LIVE/DEAD™ Fixable Near-IR Dead Cell Stain Kit, for 633 or 635 nm excitation (Life Technologies, Burlington, ON, Canada); 2% FBS/Dulbecco’s phosphate-buffered saline (DPBS) (Gibco, Life Technologies); 2% paraformaldehyde (PFA)/phosphate-buffered saline (PBS) (Avocado Research Chemicals Ltd., Heysham, UK); 96-well U-bottom culture plates (Falcon^®^, Corning, New York, NY, USA); and a LEGENDplex™ Human Inflammation Panel 1 (13-plex) with V-bottom Plate (BioLegend^®^, San Diego, CA, USA).

All samples used for HPLC were filtered through a 25 mm, 0.2 µM polyvinylidene fluoride (PVDF) Whatman UNIFLO™ syringe filter (Cytiva Life Sciences, Little Chalfont, UK).

### 2.2. Extraction and Isolation

#### 2.2.1. General Procedures

##### HPLC

Preparative reverse-phase (RP)-HPLC was performed on an Agilent 1260 Infinity II HPLC system (Agilent Technologies, Waldbronn, Germany) coupled with a diode array detector (DAD) (monitored at 214 nm, 254 nm, and 280 nm wavelengths) and equipped with a Phenomenex Synergi 10 µm Fusion-RP 80 Å (250 mm × 21.2 mm) column (Phenomenex, Torrance, CA, USA). HPLC buffer A was 0.05% (*v*/*v*) trifluoroacetic acid (TFA)/H_2_O, and buffer B was 10% H_2_O/MeCN with an additional 0.045% (*v*/*v*) TFA. Method: 4 mL/min; 0 min—10% buffer B → 80 min—50% buffer B → 82 min—90% buffer B → 92 min—90% buffer B → 95 min—10% buffer B. Peak retention times (RT) are reported in minutes based on the 254 nm chromatogram.

##### Structural Elucidation by NMR Spectroscopy

Proton (^1^H), carbon (^13^C), 2-dimensional (2D)-correlation spectroscopy (COSY), 2D heteronuclear multiple bond correlation spectroscopy (HMBC), 2D single quantum coherence spectroscopy (HSQC), and 2D nuclear Overhauser effect spectroscopy (NOESY) NMR experiments were performed on a Bruker 600 MHz AVANCE III NMR spectrometer (Bruker, Karlsruhe, Germany) equipped with a cryoprobe and utilizing IconNMR software (version 3.6.5). All experiments were run at 298 K. Chemical shifts are recorded in parts per million (ppm) with coupling constants (*J*) reported in Hertz (Hz) and multiplicities reported as singlets (s), broad singlets (bs), doublets (d), triplets (t), and multiplets (m). Processing and display of NMR data was accomplished using Mestrelab Research Mestrenova 9.0.1. All NMR spectra were run in CD_3_OD and referenced to the residual solvent peaks in the ^1^H NMR (δ = 3.31) and ^13^C NMR (δ = 49.00) spectra, respectively.

##### Mass Determination by Mass Spectrometry

Electrospray ionization (ESI) low-resolution mass spectrometry (LRMS) was performed on a Shimadzu LCMS-2020 mass spectrometer (Kyoto, Japan) in positive (ESI^+^) or negative (ESI^−^) ionisation mode at James Cook University (JCU) or the University of Wollongong (UOW). Isolated compounds were prepared for LRMS in HPLC-grade MeOH. The ion mass-to-charge (*m*/*z*) values are reported as the relative abundance compared to the base peak. The molecular ion is reported as M. All MS data were processed using Shimadzu LabSolutions software (version 5.96).

#### 2.2.2. Extract Preparation for Initial Screening

Samples were extracted based upon previously reported methods [25]. The dried aerial organs and roots (2.2 g each) of *T. hemprichii* (TH), *C. rotundata* (CR), *H. uninervis* (HU), *S. isoetifolium* (SI), and *Z. muelleri* (ZM) were macerated three times in acetone (50 mL) for 30 min. The combined washes were evaporated to dryness to generate the TH (40 mg, 7.3%), CR (30 mg, 5.5%), HU (40 mg, 7.3%), SI (30 mg, 5.5%), and ZM (30 mg, 5.5%) extracts.

#### 2.2.3. Large-Scale Preparation of *Z. muelleri* Aerial and Root Extracts

The *Z. muelleri* aerial parts (ZMA, 64.74 g) and root parts (ZMR, 27.86 g) were separated and macerated twice in acetone (aerial: 1.2 L; roots: 0.75 L) for 1 day. Both washes were pooled and evaporated to dryness to yield the ZMA (1.07 g, 1.7%) and ZMR (0.52 g, 1.9%) crude extracts.

#### 2.2.4. Purification of *Z. muelleri* Aerial Extract

The ZMA extract was purified and compounds elucidated based upon previously reported methods [25].

The ZMA extract (1.07 g) was suspended in 100 mL MeOH and extracted with hexanes (5 × 100 mL) to remove non-polar compounds, and the MeOH was evaporated to yield the MeOH fraction (115 mg). The MeOH fraction was dissolved in 10% buffer A/buffer B, filtered through a PVDF syringe filter, and subjected to RP-HPLC to yield 4-hydroxybenzoic acid (**1**) (RT = 26.208 min), luteolin (**2**) (RT = 71.001), and apigenin (**3**) (RT = 75.055).

4-Hydroxybenzoic acid (**1**)

Isolated as an amorphous white solid (0.7 mg, 0.07%). All spectral characteristics matched previously reported data [26]. ^1^H NMR (600 MHz, CD_3_OD) δ (ppm) 7.87 (d, *J* = 8.8 Hz, 2H), 6.81 (d, *J* = 8.8 Hz, 2H), not observed due to H/D-exchange (2H, COOH, ArOH); ^13^C NMR (151 MHz, CD_3_OD) δ (ppm) 170.0, 163.4, 133.0, 122.7, 116.0; LRMS (ESI^−^) *m*/*z* 137 (100%) [M−H]^−^.

Luteolin (**2**)

Isolated as an amorphous yellow solid (3.2 mg, 0.32%). Spectral characteristics matched previously reported data [27]. ^1^H NMR (600 MHz, CD_3_OD) δ (ppm) 7.40–7.37 (m, 2H), 6.91 (d, *J* = 8.1 Hz, 1H), 6.55 (s, 1H), 6.45 (d, *J* = 2.1 Hz, 1H), 6.21 (d, *J* = 2.1 Hz, 1H), not observed due to H/D-exchange (4H, ArOH); ^13^C NMR (151 MHz, CD_3_OD) δ (ppm) 183.8, 166.4, 166.0, 163.2, 159.4, 151.0, 147.0, 123.7, 120.3, 116.8, 114.2, 105.3, 103.9, 100.1, 95.0; LRMS (ESI^+^) *m*/*z* 287 (100%) [M+H]^+^.

Apigenin (**3**)

Isolated as an amorphous yellow solid (7.7 mg, 0.76%). Spectral characteristics matched previously reported data [28]. ^1^H NMR (600 MHz, CD_3_OD) δ (ppm) 7.86 (d, *J* = 8.8 Hz, 2H), 6.93 (d, *J* = 8.8 Hz, 2H), 6.60 (s, 1H), 6.46 (d, *J* = 2.1 Hz, 1H), 6.21 (d, *J* = 2.1 Hz, 1H), not observed due to H/D-exchange (3H, ArOH); ^13^C NMR (151 MHz, CD_3_OD) δ (ppm) 183.9, 166.3, 166.0, 163.2, 162.8, 159.4, 129.5, 123.3, 117.0, 105.3, 103.8, 100.1, 95.0; LRMS (ESI^+^) *m*/*z* 271 (100%) [M+H]^+^.

### 2.3. Biological Screening

#### 2.3.1. Radical Scavenging Antioxidant Assay

Using a modified literature method [29,30,31], the DPPH radical scavenging activity of each seagrass root and aerial extract was evaluated (performed in triplicate) by combining 100 μL of root and aerial extracts (and separated root or aerial extract for *Z. muelleri*) at five different concentrations (500, 250, 125, 62.5, and 32.5 μg/mL in MeOH) with 200 μL of 0.1 mM DPPH (in MeOH). Absorbances were measured using a microplate reader (SPECTROstar^®^ Omega, BMG Labtech, Sydney, Australia) at 517 nm after 1 h of incubation in the dark condition at room temperature. Methanol was used as a blank, and gallic acid was used as a standard antioxidant compound and was tested at the same five concentrations as the plant extracts. The percentage of DPPH radical scavenged by the sample was calculated using the formula:

$$\%\;DPPH\;radical\;scavenged=(\frac{A_{c}-A_{1}}{A_{c}})\times 100\%$$

where *A*_1_ = absorbance of the sample (at 517 nm), *A_c_* = absorbance of the control (at 517 nm).

All antioxidant assay data were visualized using GraphPad Prism 10.2.2. The half maximal inhibitory concentrations (IC_50_) values were measured at the concentration of 50% DPPH radical inhibition. Negative DPPH radical scavenging percentages were normalised to 0%.

#### 2.3.2. Anti-Inflammatory Activity and Quantification

All cell viability and anti-inflammatory screening procedures were performed according to previously reported methods [25,32].

##### PBMC Collection and Culture Conditions

The ethics approval (H8523) for this assay was granted by the Human Research Ethics Committee of James Cook University in 2023. Blood from two healthy donors was supplied by the Red Cross Lifeblood, Australia. The human peripheral blood mononuclear cells (PBMCs) were separated from the blood samples using a Ficoll-Paque PLUS density gradient method according to the manufacturer’s instructions and cryopreserved in filtered FBS containing 10% DMSO.

##### Sample Treatment

According to a previously reported method [25,32], the PBMCs were stimulated with lipopolysaccharide (LPS, 10 ng/mL) and treated with extracts simultaneously. For all tests, 1 × 10^6^ cells in 100 μL of R-10 media (RPMI-1640, containing 10% heat-inactivated FBS, 100 U/mL penicillin, and 100 μg/mL streptomycin) were seeded into the wells of 96-well U-bottom culture plates. Stimulated PBMCs were treated in triplicate with the ZMA crude extract (100 μg/mL in cell culture media with 0.5% DMSO). Culture plates were incubated overnight at 37 °C in a 5% CO_2_ incubator. Following overnight incubation, plates were centrifuged (277× *g*, 4 °C, 5 min), and the culture supernatants were collected and kept in a −80 °C freezer until further analysis for cytokine profile. The PBMCs left in the culture plate were immediately stained to determine the effect of the ZM crude extract on cell viability, as described in the subsequent section.

##### Determination of Cell Viability

After collecting the supernatant, cells were stained with viability dye (LIVE/DEAD™ Fixable Near-IR Dead Cell Stain Kit) following the manufacturer’s instructions. Stained cells were rinsed twice with 200 μL 2% FBS/Dulbecco’s phosphate-buffered saline after 30 min (kept on ice under the dark condition). Cells were then resuspended in 2% PFA/PBS (100 μL) and analysed by flow cytometry (LSRFortessa™ X20, BD Biosciences, Franklin Lakes, NJ, USA), and the data was examined using the FlowJo software (version 10.8.1). Before determining the %Live/Dead dye-positive cells, doublets were excluded from the analysis by gating forward scatter height (FSC-H) versus forward scatter area (FSC-A). After obtaining the cell viability results, the culture supernatant was further analysed to quantify the amount of pro-inflammatory cytokines.

##### Quantification of Proinflammatory Cytokines

On the day of the experiment, PBMCs culture supernatant from the −80 °C freezer was allowed to thaw at room temperature. The customised LEGENDplex^TM^ human inflammation panel multi-analyte flow assay kit was utilised to perform the assay following the manufacturer’s instructions. This panel allows simultaneous quantification of 13 human inflammatory cytokines, including TNF-α, interferon alpha (IFN-α), IFN-γ, monocyte chemoattractant protein-1 (MCP-1), IL-1β, IL-6, IL-8 (CXCL-8), IL-12p70, IL-10, IL-17A, IL-18, IL-33, and IL-23. All data were acquired on a flow cytometer and exported in FCS (flow cytometry standard) format. For the determination of cytokine concentrations/profiles, flow cytometry data files in flow cytometry standard (FCS) format were analysed using the cloud-based BioLegend^®^ Software (version 2023-02-15, Qognit, Inc., San Carlos, CA, USA). All tests were performed in triplicate and are expressed as mean ± standard deviation (SD). Cytokine levels were normalised so LPS treatment groups equalled 100%. All data were analysed statistically and visualised using GraphPad Prism (10.2.2). Statistical analysis was performed using a Welch’s *t*-test, where ns = not significant, * = *p* < 0.05, ** = *p* < 0.01, *** = *p* < 0.001, **** = *p* < 0.0001.

## 3. Results

### 3.1. DPPH Radical Scavenging Activity of Seagrasses

**Figure 2:**
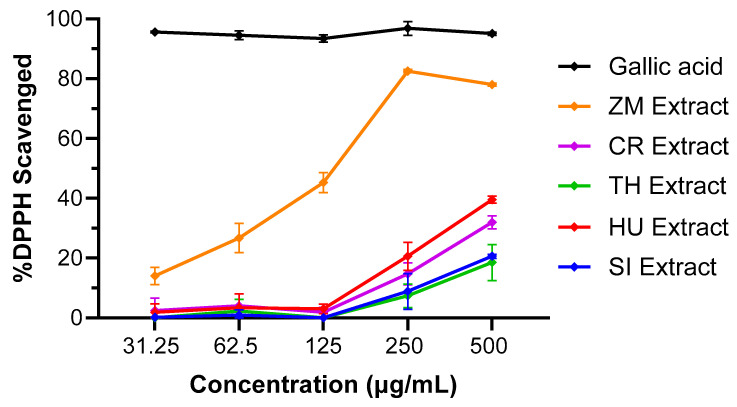
The 1,1-diphenyl-2-picrylhydrazyl (DPPH) free radical scavenging activity of five seagrass aerial and root tissues’ crude extract (31.25–500 μg/mL concentrations). The data expressed represent mean ± standard deviation (SD) from two independent experiments performed in triplicate (n = 6), visualized using GraphPad Prism 10.2.2. Gallic acid was included as a standard antioxidant compound. Plant species abbreviations are as follows: SI—*S. isoetifolium*; HU—*H. uninervis*; TH—*T. hemprichii*; CR—*C. rotundata*; ZM—*Z. muelleri*.

A DPPH radical scavenging antioxidant assay [33] of the homogenised root and aerial parts of *Halodule uninervis*, *Cymodocea rotundata*, *Syringodium isoetifolium*, *Thalassia hemprichii*, and *Zostera muelleri* revealed that the *Z. muelleri* extract had the best antioxidant activity of the five seagrass species tested, with 83% of DPPH radical scavenged at 250 μg/mL and an IC_50_ value of 138 µg/mL compared to the other seagrasses tested, which all had less than 40% scavenging effect at 500 μg/mL concentration (Figure 2, Table 1).

### 3.2. In-Depth Investigation of Z. muelleri Aerial and Root Parts

**Figure 3:**
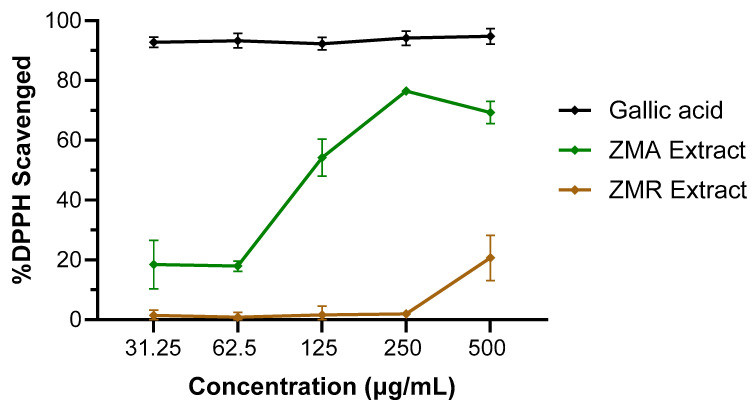
The DPPH-free radical scavenging activity by aerial or root crude extracts (31.25–500 μg/mL concentrations) of *Z. muelleri*. The data expressed represent mean ± SD from two independent experiments performed in triplicate (n = 6), visualized using GraphPad Prism 10.2.2. Gallic acid was included as a standard antioxidant compound for comparison.

Given the higher antioxidant capacity of *Z. muelleri* aerial and root extract relative to the other seagrasses and the lack of studies performed in this species previously, additional oven-dried whole plant materials were separated into aerial (ZMA) and root (ZMR) bulk samples, which were macerated in acetone to produce crude extracts. Separate DPPH radical scavenging assays were performed on the root and aerial extracts, which showed that leaves had a better DPPH radical scavenging capacity of 75% (at 250 µg/mL) and an IC_50_ value of 119 μg/mL compared to roots, which had a maximum DPPH radical scavenging capacity of 20% at 500 μg/mL (Figure 3).

**Figure 4:**
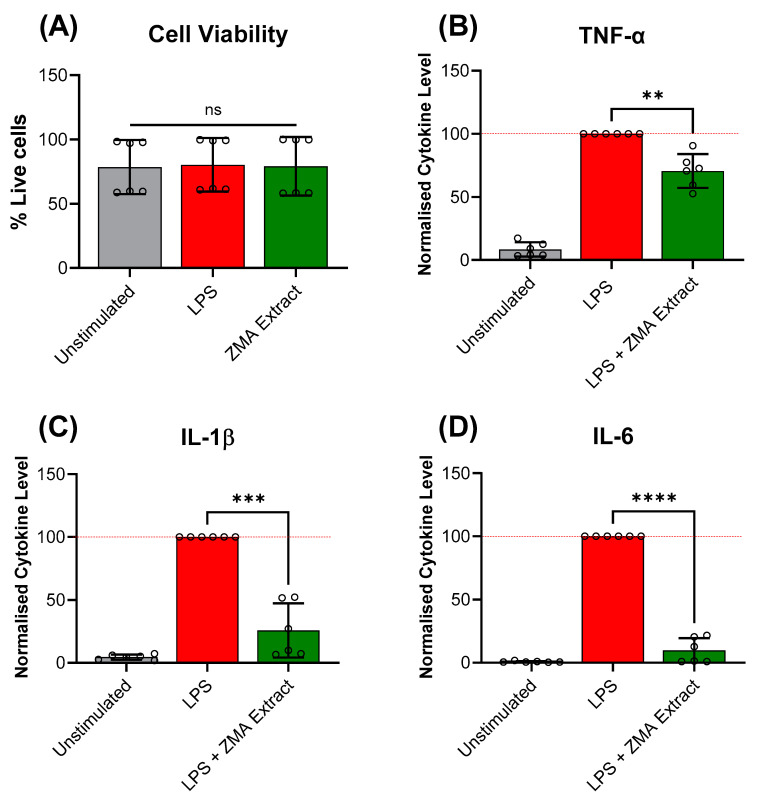
The (**A**) cell viability of human peripheral blood mononuclear cells (PBMCs) with aerial crude extract of *Z. muelleri* at a 100 μg/mL concentration after overnight incubation; and the normalized cytokine levels of (**B**) tumour necrosis factor alpha (TNF-α) (**C**) interleukin (IL)-1β, and (**D**) IL-6 released by the PBMCs stimulated with bacterial lipopolysaccharide (LPS). Bar plots show the mean ± SD from two independent experiments performed in triplicate (n = 6), visualised using GraphPad Prism 10.2.2. For cytokine plots, LPS treatment groups were normalized to 100%. Statistical analysis was performed using a Welch’s *t*-test, where ns = not significant, ** = *p* < 0.01, *** = *p* < 0.001, **** = *p* < 0.0001.

Since the aerial extract of *Z. muelleri* had a higher antioxidant capacity, its anti-inflammatory properties were subsequently quantified using a human PBMC assay and LEGENDplex cytokine analysis. A cell viability assay demonstrated no statistical significance in the amount of cell death between the unstimulated and ZMA crude extract treatment groups, indicating the extract was not toxic to the PBMCs at 100 μg/mL (Figure 4A). Analysis of the LPS-stimulated PBMCs culture supernatant revealed that treatment with ZMA crude extract significantly reduced the levels of TNF-α, IL-1β, and IL-6 released by the LPS-stimulated PBMCs relative to the LPS control by 29%, 74%, and 90%, respectively (Figure 4B–D, Table 2), while no response was observed for the remaining cytokines tested.

### 3.3. Bioactive Compounds Isolated Using HPLC

**Figure 5:**
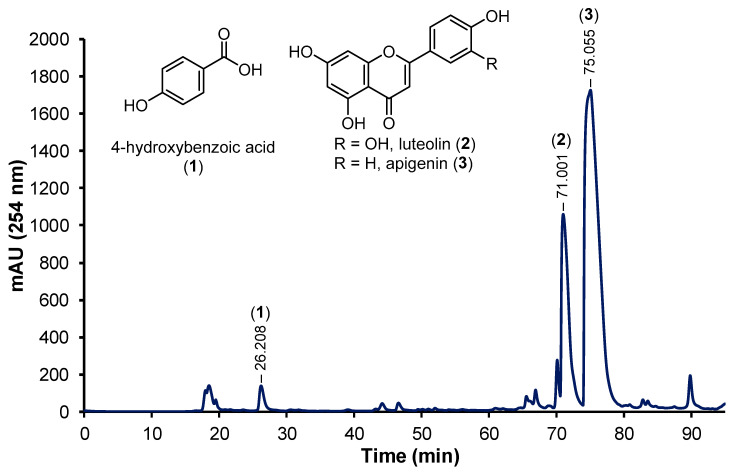
The preparative high-performance liquid chromatography (HPLC) chromatogram (254 nm) of the methanol (MeOH) fraction of the *Z. muelleri* aerial extract and chemical structures of isolated compounds 4-hydroxybenzoic acid (**1**), luteolin (**2**), and apigenin (**3**).

**Figure 6:**
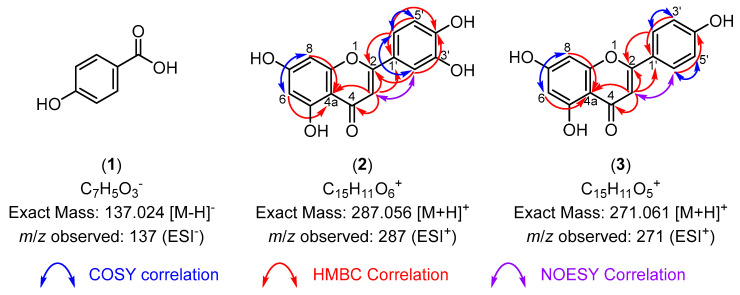
The key two-dimensional (2D) nuclear magnetic resonance (NMR) correlations and low-resolution mass spectrometry (LRMS) data used for the structural elucidation of compounds (**1**)–(**3**). No 2D NMR spectroscopy was performed on 4-hydroxybenzoic acid (**1**) due to its structural simplicity.

To isolate the potential active principles responsible for the antioxidant and anti-inflammatory activities observed in the aerial extract of *Z. muelleri*, the ZMA extract was dissolved in MeOH and washed several times with hexanes. The MeOH fraction was purified by reverse-phase high-performance liquid chromatography (RP-HPLC), which led to the isolation of 4-hydroxybenzoic acid (**1**), luteolin (**2**), and apigenin (**3**) (Figure 5). The structure of 4-hydroxybenzoic acid (**1**) was determined by comparison of the NMR spectrum (see Supporting Information Figures S1 and S2) to previously reported spectra [26] and the observation of a peak at *m*/*z* 137 (ESI^−^) in the LRMS, which was assigned as the [M−H]^−^ ion of (**1**) (Figure 6).

The structures of luteolin (**2**) and apigenin (**3**) were elucidated using 2D NMR spectra (see Supporting Information Figures S3–S14), particularly the HMBC spectra. The observation of key correlations between H6, H8, H3, and C4a was used to assign the flavonoid core structure (Figure 6). The observation of additional HMBC correlations between H3 and carbonyl C4 and phenyl substituent carbon C1′ indicated these were flavone derivatives (Figure 6). The phenyl substituents of luteolin (**2**) and apigenin (**3**) were assigned based on COSY correlations between the H2, H3, and H6 protons, which indicated the presence of a 1,2,4-trisubstituted and 1,4-disubstituted aromatic system, respectively. Analysis of the LRMS (ESI^+^) of luteolin (**2**) and apigenin (**3**) showed peaks at *m*/*z* 287 and 271, respectively, which were assigned as the [M+H]^+^ ions in both cases (Figure 6). These structures showed identical spectral information to that previously reported data for luteolin [27] and apigenin [28], unambiguously confirming the structures of luteolin (**2**) and apigenin (**3**).

## 4. Discussion

Seagrasses grow in marine conditions and produce secondary metabolites for various ecological functions ranging from feeding deterrence [34] to protection from ultraviolet (UV) radiation [35]. In defence against pathogens and predators, numerous species of seagrasses produce a wide array of phenolic compounds, thus gaining attention for applications in nutrition, medicine, and cosmetics [36]. While considerable phytochemical research has been conducted, a gap persists in our knowledge of seagrass secondary metabolites [34].

The DPPH radical scavenging antioxidant activity of *C. rotundata*, *T. hemprichii*, and *H. uninvervis* (IC_50_ > 500 µg/mL, Table 1) in the DPPH assays were significantly weaker compared to previously reported values (IC_50_ = 214.68 [37], 123.72 [37], and 4.0 µg/mL [38], respectively) while those of *S. isoetifolium* (IC_50_ > 500 µg/mL) correlated with previous values (520.91 µg/mL [37]). This reduced antioxidant activity may be the result of seasonality, with samples collected in this study close to winter instead of summer, which has been reported to result in reduced antioxidant production in seagrasses previously due to the reduced ecological stress (lower temperatures and light intensities) and therefore do not need to produce protective secondary metabolites in large quantities [39].

To the best of our knowledge, this study represents the first investigation of the antioxidant and anti-inflammatory activity of *Z. muelleri*, which showed good DPPH radical scavenging activities for its aerial and root (138 µg/mL, Table 1) and aerial (119 µg/mL, Table 2) extracts. This potent antioxidant activity observed in the *Z. muelleri* extracts suggested it may also exert anti-inflammatory effects due to the high oxidative stress inherent in inflammatory responses [40]. These suspicions were confirmed by the significant reduction of TNF-α, IL-1β, and IL-6 cytokine levels in LPS-stimulated PBMCs culture supernatant (29%, 74%, and 90%, respectively, Figure 4). This significant reduction in IL-1β and IL-6 observed coupled with the established correlation between acne vulgaris and IL-1β [41] and IL-6 [42,43] cytokine levels suggest *Z. muelleri* extracts could be developed into an effective treatment for acne and could be a potential source for the discovery of new anti-inflammatory lead compounds.

As part of a search for new anti-inflammatory lead compounds, the isolation of 4-hydroxybenzoic acid (**1**), luteolin (**2**), and apigenin (**3**) from the ZMA extract represented the first isolation of small molecules from *Z. muelleri* as only qualitative phytochemical analyses [13,14] and metabolomic studies [44,45,46,47] had been reported previously. 4-Hydroxybenzoic acid (**1**) is believed to perform anti-bacterial and anti-inflammatory functions in plants [48] and has been detected in several seagrasses previously, including *Halodule pinifolia* [49], *Posidonia oceanica* [50], *Syringodium isoetifolium* [51], *Thalassia hemprichii* [52], and *Thalassia testudinum* [53].

Luteolin (**2**) and apigenin (**3**) are closely related simple flavone derivatives, the latter known for their broad bioactivities [54]. Both of these flavones are extremely common in many plant species and are known to protect plants from UV radiation [55,56] and have anti-microbial, antioxidant, and anti-inflammatory properties [55,56,57,58,59]. Both luteolin (**2**) and apigenin (**3**) have been reported to exert their antioxidant and inflammatory properties through the deactivation of the nuclear factor kappa-light-chain-enhancer of activated B cells (NF-κB) pathway, modulation of the MAPK signalling pathways and inhibition of many inflammatory cytokines including TNF-α, IL-1β, IL-6, IL-8, IL-17, and nitric oxide (NO) in a host of in vitro, ex vivo, and in vivo models [59,60,61,62,63]. Given that both luteolin (**2**) and apigenin (**3**) were the major components isolated and the extensive literature on their potent bioactivities, this suggests these compounds are the major bioactive components contributing to the observed antioxidant and anti-inflammatory activities of the *Z. muelleri* aerial extract.

These results provide a strong case for further research into using seagrasses to treat inflammatory conditions. For example, the UV protection potential of luteolin (**2**) and apigenin (**3**) [55,56], coupled with their anti-inflammatory properties in keratinocytes and fibroblasts, [60,64,65] would make seagrass extracts ideal candidates for the production of topical treatments for the treatment of inflammatory skin conditions such as acne or eczema. In addition, it has been established that the NF-κB pathway and TNF-α, IL-1β, and IL-6 cytokine levels are significantly upregulated within 24 h of experiencing sunburn and remain elevated four days after UV irradiation [66]. Given the significant reduction in these pro-inflammatory cytokines observed in the ZMA extract, its antioxidant activity, and its potential UV protective properties, with further research there is the potential for *Z. muelleri* extracts to be developed into all-in-one sunscreen and sunburn-soothing ointments.

The key limiting factors of this study were insufficient access to *Z. muelleri* plant materials, which only allowed for the isolation of abundant, known active principles and that the anti-inflammatory investigation of the ZMA extract was performed with a low sample size (n = 6). Therefore, further research with more plant material is required to isolate minor secondary metabolites, and additional screening of the ZMA extract is also needed to confirm these promising preliminary results and justify further study of in vivo models of inflammation. Other research has suggested seagrasses could be consumed for various gastrointestinal ailments [36], with several species also possessing anti-diabetic properties [17,67,68], highlighting additional avenues for future inquiry and showcasing the potential versatility of seagrasses in their uses and the need for further investigation into their seemingly endless list of applications.

## 5. Conclusions

The investigation of the bioactivities of the five selected seagrasses—*Zostera muelleri*, *Halodule uninervis*, *Cymodocea rotundata*, *Syringodium isoetifolium*, and *Thalassia hemprichii*—showed that acetone extracts of *Z. muelleri* had the most potent antioxidant activity in a DPPH free radical scavenging assay. The aerial extract of *Z. muelleri* possessed significantly higher antioxidant activity than that of the roots. It was also shown to significantly reduce the levels of key inflammatory cytokines TNF-α, IL-1β, and IL-6 in human PBMCs. Purification of the *Z. muelleri* aerial extract led to the isolation of 4-hydroxybenzoic acid (**1**), luteolin (**2**), and apigenin (**3**), which were suggested to be the major anti-inflammatory components of the aerial extract. These preliminary results emphasise the potential utility of seagrasses in the treatment of inflammatory conditions and highlight the need for further research with additional *Z. muelleri* plant material to isolate other potential active principles, confirm the anti-inflammatory properties of the ZMA extract in vivo, and evaluate this extract for utility as a topical treatment for inflammatory skin conditions and as a sunscreen.

## Figures and Tables

**Table 1:** Summary of the DPPH radical scavenging activities and half maximal inhibitory concentration (IC_50_) values of the five seagrass aerial and root extracts. * Radical scavenging activity observed at 500 µg/mL. ^†^ Reported as the mean ± SD.

Sample	%DPPH Radical Scavenging Activity *^†^	IC_50_ (µg/mL)
Gallic acid	95.1 ± 0.5%	<31.25
ZM Extract	78.0 ± 0.5%	138
CR Extract	32.0 ± 2.2%	>500
TH Extract	18.5 ± 6.0%	>500
HU Extract	39.6 ± 1.2%	>500
SI Extract	20.6 ± 0.7%	>500

**Table 2:** Summary of the antioxidant DPPH radical scavenging activities and IC_50_ values and the anti-inflammatory PBMC assay normalised cytokine levels of the *Z. muelleri* aerial (ZMA) and root (ZMR) extracts. * Radical scavenging activity observed at 500 µg/mL. ^†^ Reported as the mean ± SD.

Sample	%DPPH Radical Scavenging Activity *^†^	IC_50_ (µg/mL)	Normalised Cytokine Levels ^†^		
			TNF-α	IL-1β	IL-6
Gallic acid	94.8 ± 2.6%	<31.25	–	–	–
ZMA Extract	69.3 ± 3.7%	119	70.5 ± 13.4	25.8 ± 21.6	9.6 ± 9.9
ZMR Extract	20.7 ± 7.5%	>500	–	–	–

## Data Availability

The data presented in this study is available in the electronic Supplementary Information of this article.

## Supplementary Materials

The following supporting information can be downloaded at: https://www.mdpi.com/article/10.3390/life14060710/s1: Figure S1—The ^1^H NMR spectrum of 4-hydroxybenzoic acid (**1**); Figure S2—The ^13^C NMR spectrum of 4-hydroxybenzoic acid (**1**); Figure S3—The ^1^H NMR spectrum of luteolin (**2**); Figure S4—The ^13^C NMR spectrum of luteolin (**2)**; Figure S5—The 2D COSY spectrum of luteolin (**2**); Figure S6—The 2D HMBC spectrum of luteolin (**2**); Figure S7—The 2D HSQC spectrum of luteolin (**2**); Figure S8—The 2D NOESY spectrum of luteolin (**2**); Figure S9—The ^1^H NMR spectrum of apigenin (**3**); Figure S10—The ^13^C NMR spectrum of apigenin (**3**); Figure S11—The 2D COSY spectrum of apigenin (**3**); Figure S12—The 2D HMBC spectrum of apigenin (**3**); Figure S13—The 2D HSQC spectrum of apigenin (**3**); Figure S14—The 2D NOESY spectrum of apigenin (**3**); the LRMS data for 4-hydroxybenzoic acid (**1**), luteolin (**2**) and apigenin (**3**); GraphPad Prism files of antioxidant and anti-inflammatory assays and a preparative HPLC report.
