# Supplementary material for: The In Vitro Antioxidant and Anti-Inflammatory Activities of Selected Australian Seagrasses
Source: Life (Basel). 2024 May 30;14(6):710. doi: 10.3390/life14060710 (PMC11205046; doi:10.3390/life14060710)

==== Shimadzu Labsolutions Data Report ====

Sample ID : Keller
Data Filename : SA MP1.lcd
Date Acquired :15/04/2024 3:25:19 PM

<Chromatogram>

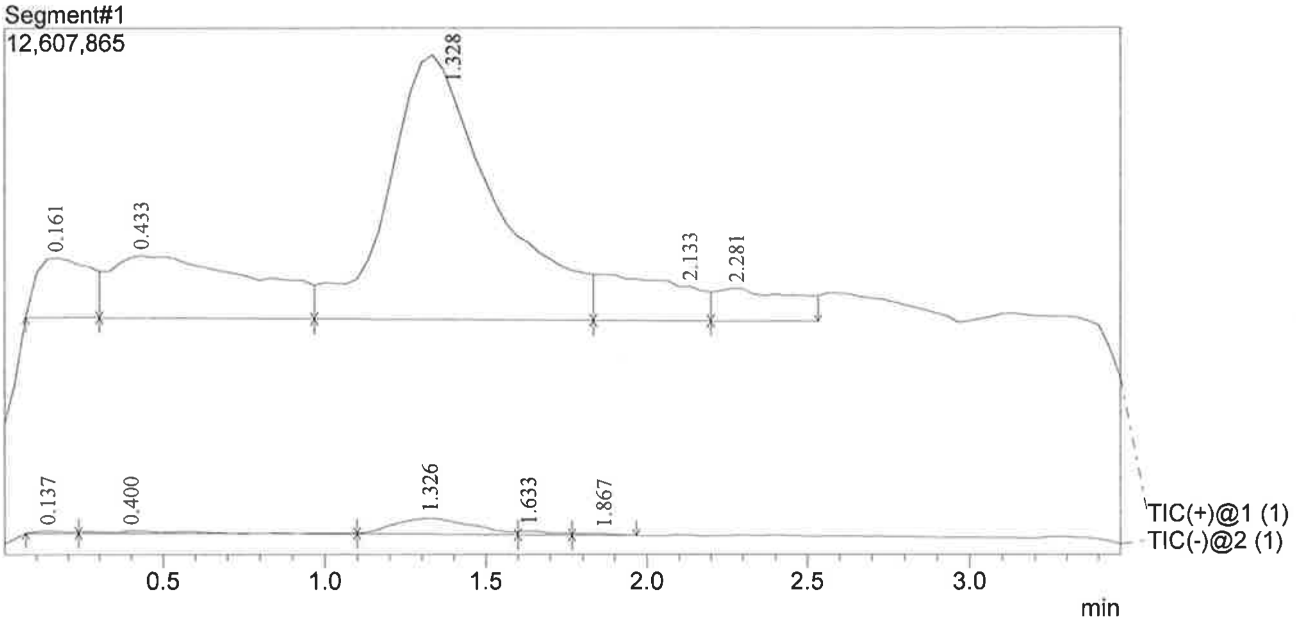

<Spectrum>

R.Time:----(Scan#:----)
MassPeaks:662 BasePeak:87(190845)
Spectrum Mode:Averaged 1.133-1.467(69-89)
BG Mode:Peak Start 0.467(29) Polarity:Positive Segment 1 - Event 1

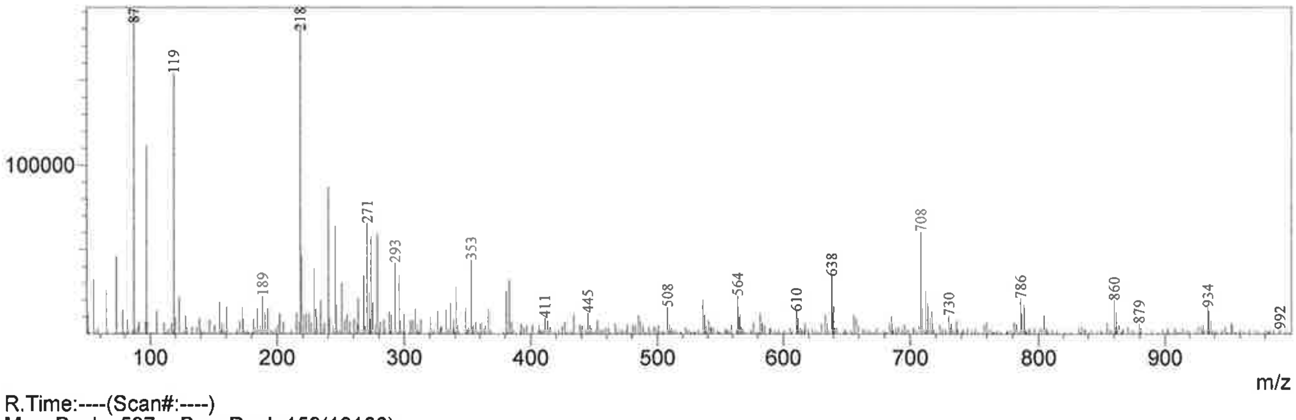

R.Time:----(Scan#:----)
MassPeaks:597 BasePeak:153(19100)
Spectrum Mode:Averaged 1.149-1.483(70-90)
BG Mode:Peak Start 0.483(30) Polarity:Negative Segment 1 - Event 2

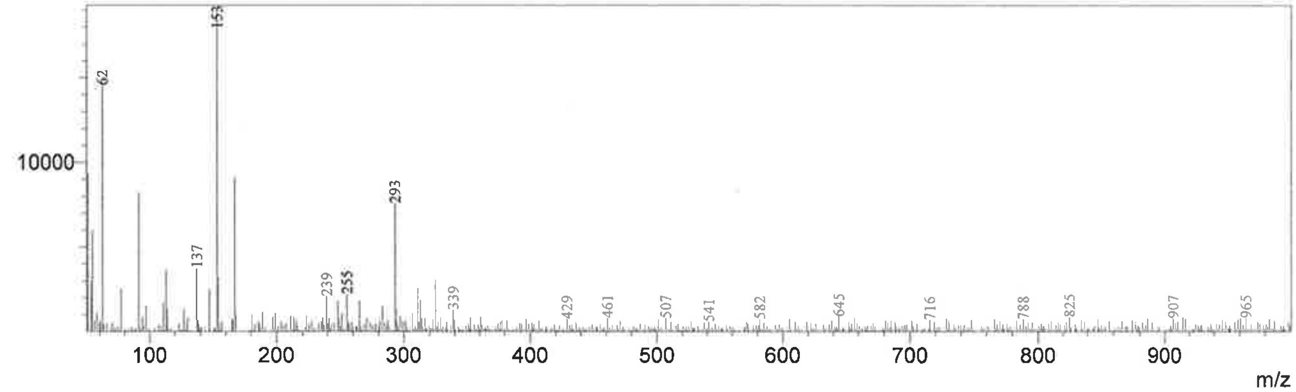

Supplement: Supplementary file 1 [file life-14-00710-s001.zip › LRMS 4-hydroxybenzoic acid (1).pdf]
