# Supplementary material for: The In Vitro Antioxidant and Anti-Inflammatory Activities of Selected Australian Seagrasses
Source: Life (Basel). 2024 May 30;14(6):710. doi: 10.3390/life14060710 (PMC11205046; doi:10.3390/life14060710)

==== Shimadzu Labsolutions Data Report ====

Sample ID	:	Date Acquired	:10/30/2023 10:38:20 AM
Data Filename	: Mara Zm MeOH Leaf HPLC D.lcd		

<Chromatogram>

Segment#1

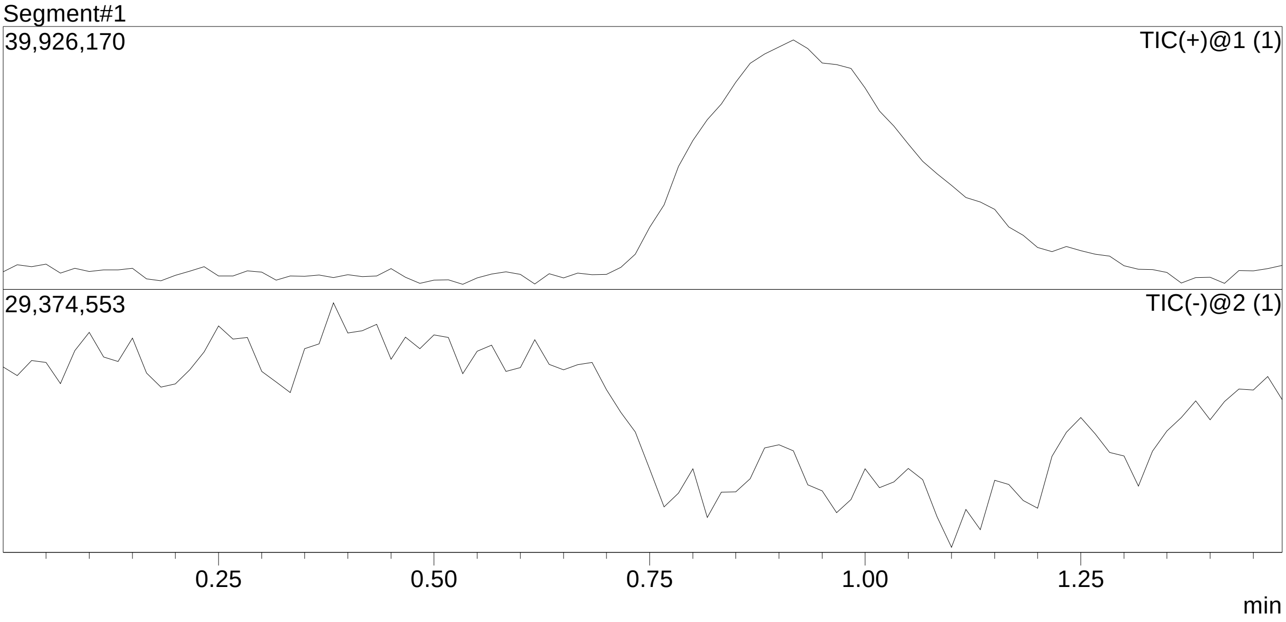

<Spectrum>

R.Time:----(Scan#:----)
MassPeaks:8 BasePeak:271.064(2222541)
Spectrum Mode:Averaged 0.750-1.083(91-131)
BG Mode:Averaged 0.067-0.367(9-45) Polarity:Positive Segment 1 - Event 1

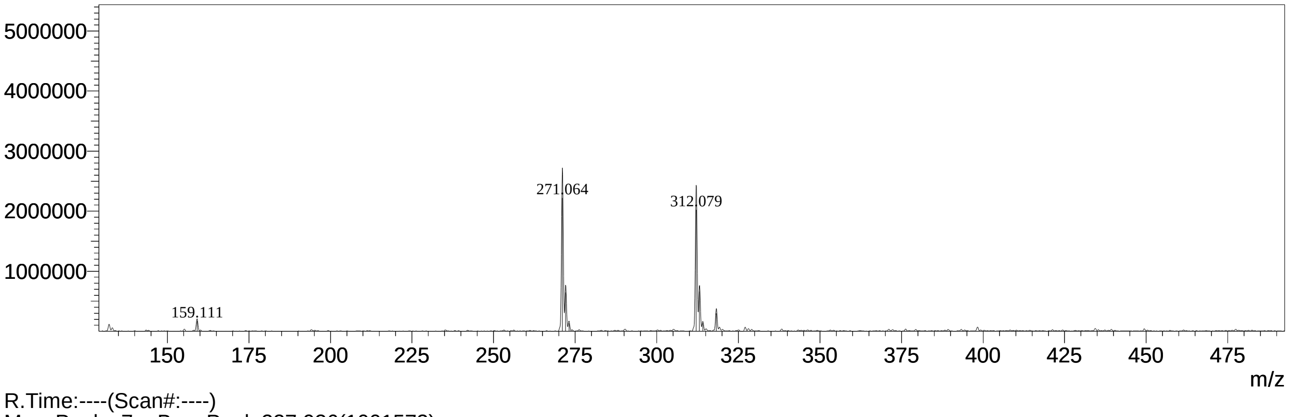

R.Time:----(Scan#:----)
MassPeaks:7 BasePeak:227.936(1001573)
Spectrum Mode:Averaged 0.757-1.090(92-132)
BG Mode:Averaged 0.073-0.373(10-46) Polarity:Negative Segment 1 - Event 2

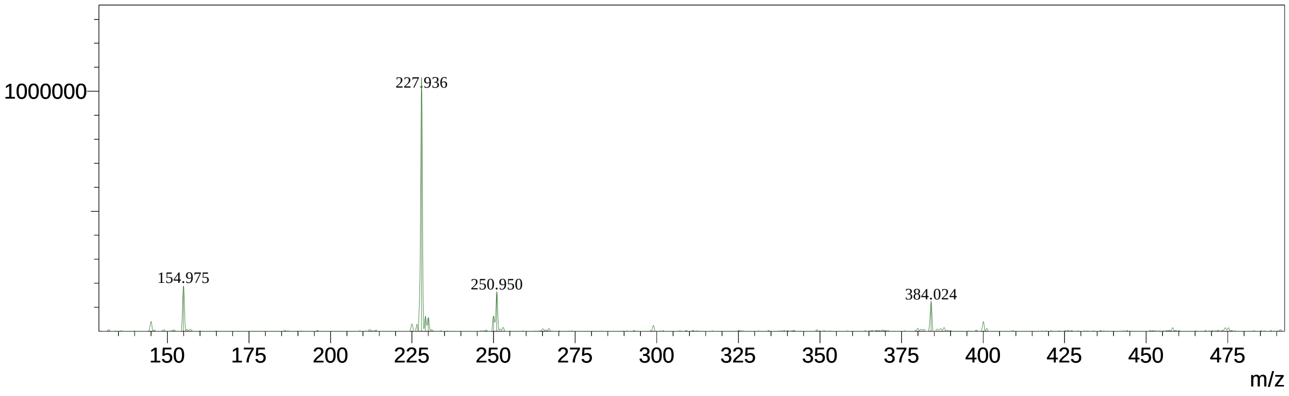

R.Time:----(Scan#:----)
MassPeaks:8 BasePeak:271.064(2222541)
Spectrum Mode:Averaged 0.750-1.083(91-131)
BG Mode:Averaged 0.067-0.367(9-45) Polarity:Positive Segment 1 - Event 1

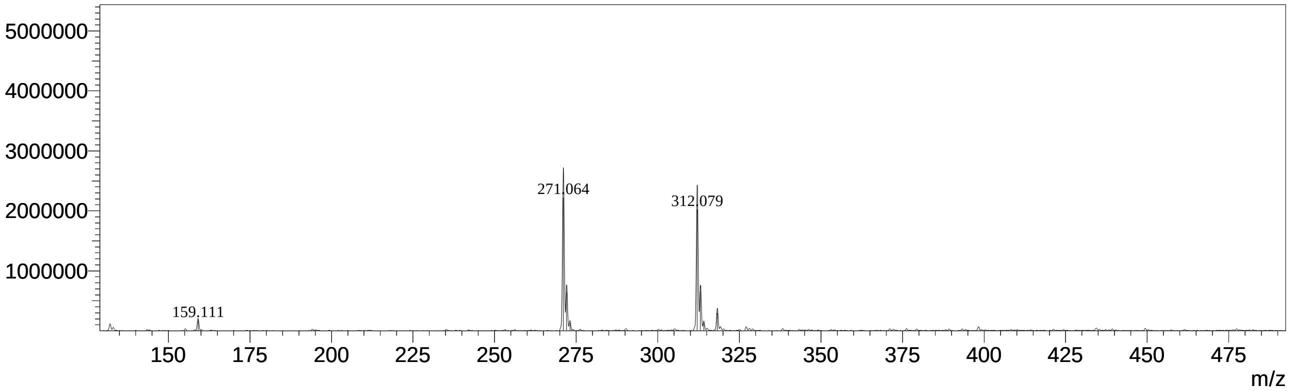

R.Time:----(Scan#:----)
MassPeaks:7 BasePeak:227.936(1001573)
Spectrum Mode:Averaged 0.757-1.090(92-132)
BG Mode:Averaged 0.073-0.373(10-46) Polarity:Negative Segment 1 - Event 2

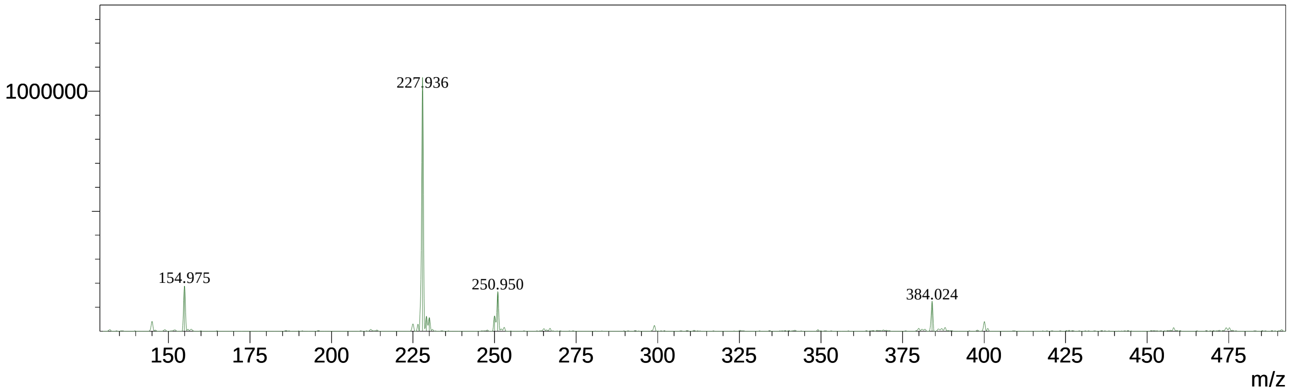

Supplement: Supplementary file 1 [file life-14-00710-s001.zip › LRMS apigenin (3).pdf]
