# Supplementary material for: The In Vitro Antioxidant and Anti-Inflammatory Activities of Selected Australian Seagrasses
Source: Life (Basel). 2024 May 30;14(6):710. doi: 10.3390/life14060710 (PMC11205046; doi:10.3390/life14060710)

==== Shimadzu Labsolutions Data Report ====

Sample ID	:	Date Acquired	:10/30/2023 10:36:11 AM
Data Filename	: Mara Zm MeOH Leaf HPLC C.lcd		

<Chromatogram>

Segment#1

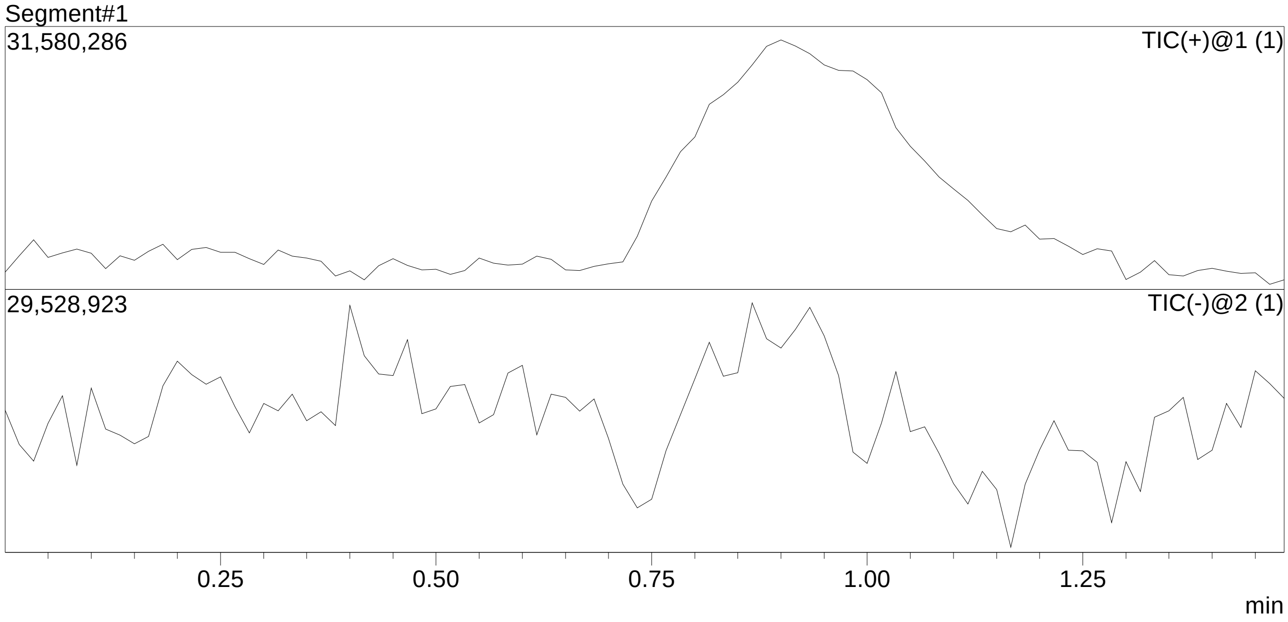

<Spectrum>

R.Time:----(Scan#:----)
MassPeaks:12 BasePeak:287.039(931276)
Spectrum Mode:Averaged 0.767-1.033(93-125)
BG Mode:Averaged 0.100-0.367(13-45) Polarity:Positive Segment 1 - Event 1

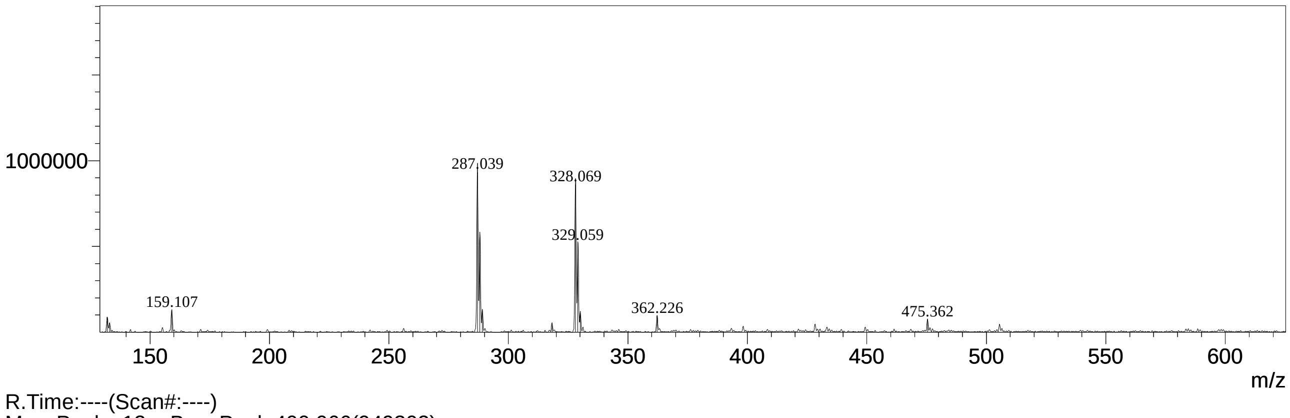

R.Time:----(Scan#:----)
MassPeaks:12 BasePeak:400.066(949302)
Spectrum Mode:Averaged 0.773-1.040(94-126)
BG Mode:Averaged 0.107-0.373(14-46) Polarity:Negative Segment 1 - Event 2

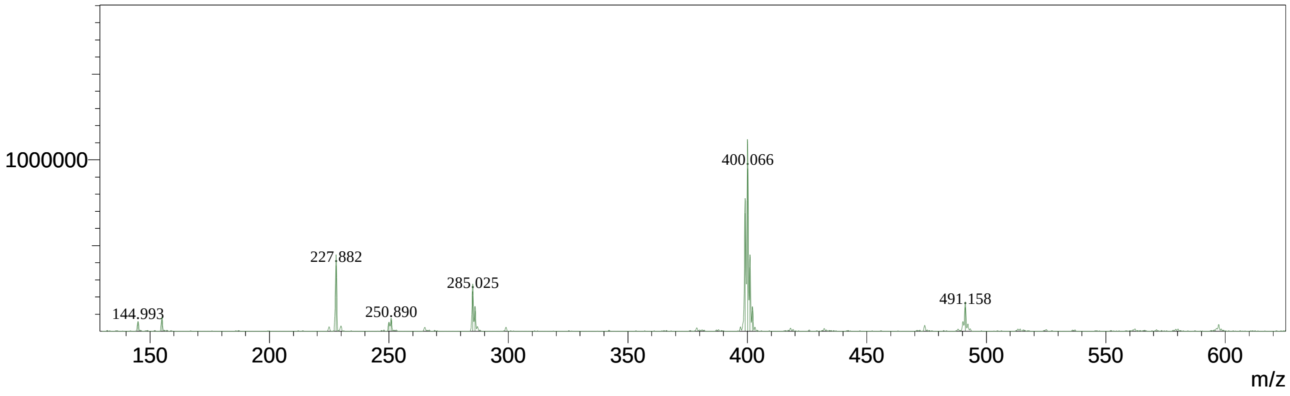

R.Time:----(Scan#:----)
MassPeaks:12 BasePeak:287.039(931276)
Spectrum Mode:Averaged 0.767-1.033(93-125)
BG Mode:Averaged 0.100-0.367(13-45) Polarity:Positive Segment 1 - Event 1

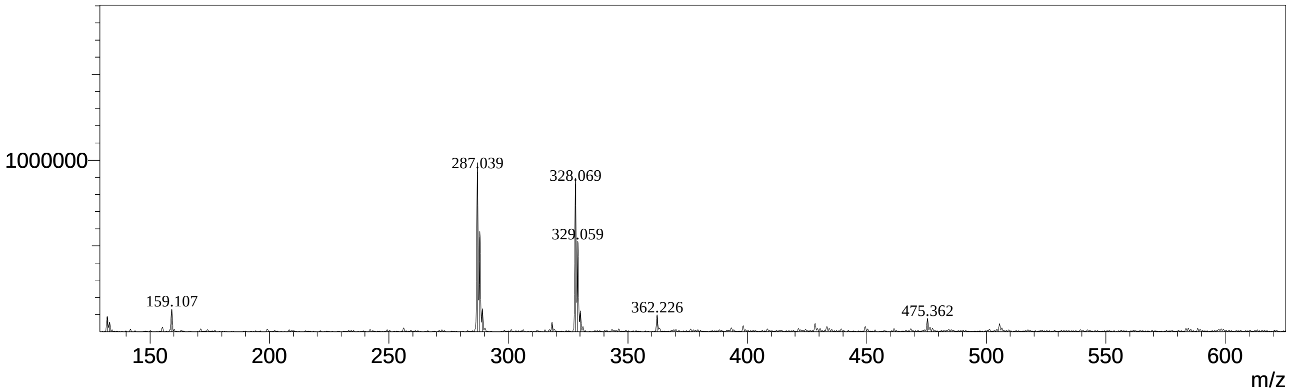

R.Time:----(Scan#:----)
MassPeaks:12 BasePeak:400.066(949302)
Spectrum Mode:Averaged 0.773-1.040(94-126)
BG Mode:Averaged 0.107-0.373(14-46) Polarity:Negative Segment 1 - Event 2

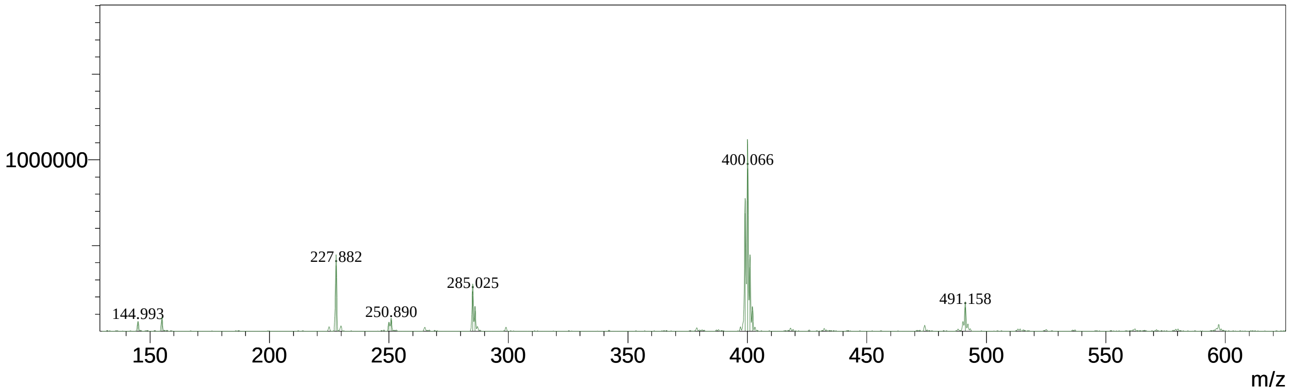

Supplement: Supplementary file 1 [file life-14-00710-s001.zip › LRMS luteolin (2).pdf]
