# Supplementary material for: The In Vitro Antioxidant and Anti-Inflammatory Activities of Selected Australian Seagrasses
Source: Life (Basel). 2024 May 30;14(6):710. doi: 10.3390/life14060710 (PMC11205046; doi:10.3390/life14060710)
Supplement: Supplementary file 1 [file life-14-00710-s001.zip › Preparative HPLC Report.pdf]

Data File C:\Chem32\1\Data\Mara\Mara 2023-10-17 15-37-38\Mara Zostera Leaf MeOH Run 4.D
Sample Name: Mara Zostera Leaf MeOH Run 4

=====================================================================

| | | | |
|---|---|---|---|
| Acq. Operator | : SYSTEM | Seq. Line : | 1 |
| Acq. Instrument | : HPLC3 | Location : | - |
| Injection Date | : 17/10/23 3:38:30 PM | Inj : | 1 |
| | | Inj Volume : | No inj |

Method : C:\Chem32\1\Data\Mara\Mara 2023-10-17 15-37-38\MC 4mL 10% IC20 50%_80M 90%_82M IC10.M (Sequence Method)

Last changed : 17/10/23 3:37:26 PM by SYSTEM

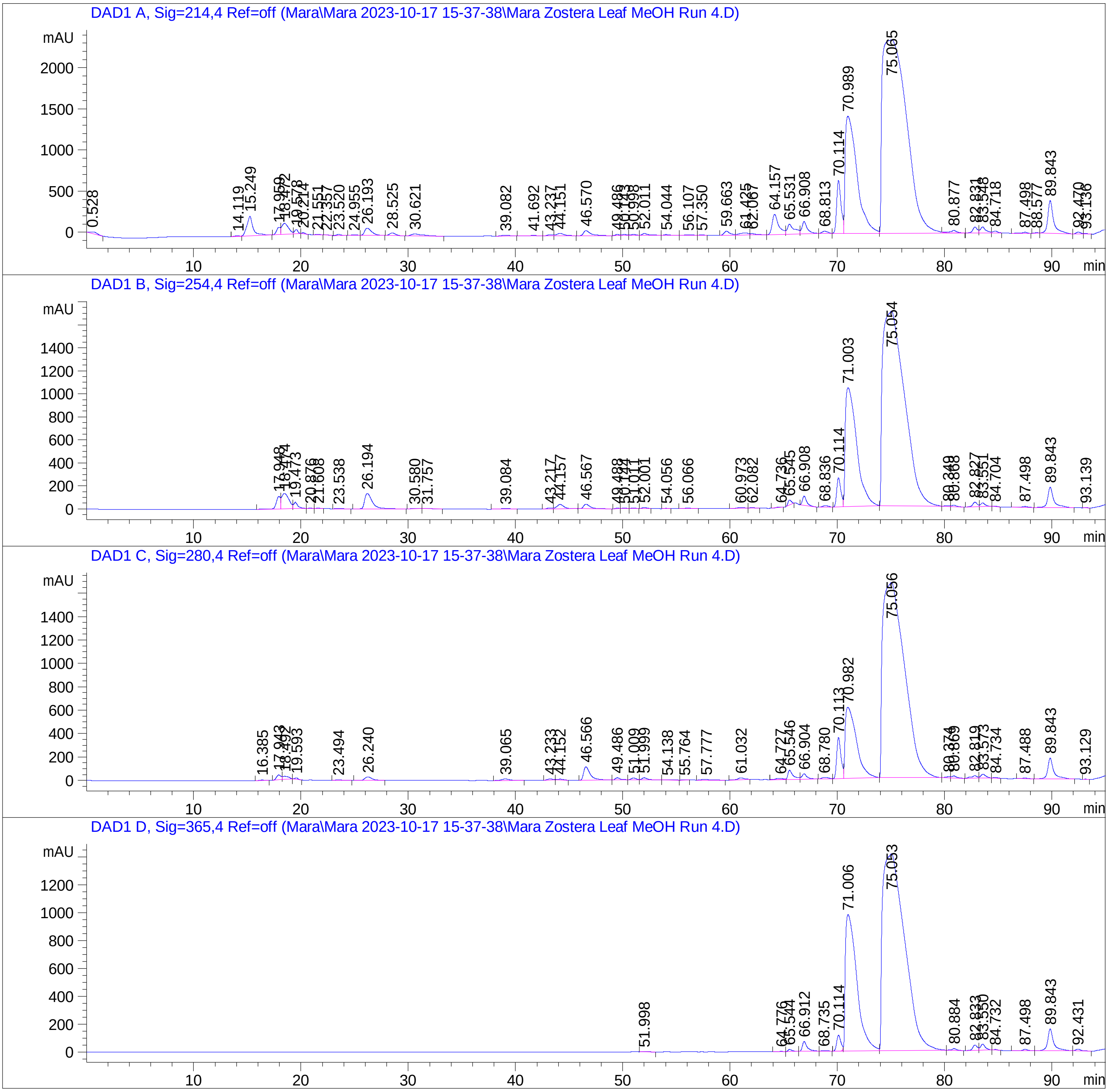


=====================================================================

Fraction Information

=====================================================================

No Fractions found.

=====================================================================

=====================================================================

=====================================================================

Area Percent Report

=====================================================================

Sorted By : Signal
Multiplier : 1.0000
Dilution : 1.0000
Use Multiplier & Dilution Factor with ISTDs

Signal 1: DAD1 A, Sig=214,4 Ref=off

| Peak # | RetTime [min] | Type | Width [min] | Area [mAU*s] | Height [mAU] | Area % |
|---|---|---|---|---|---|---|
| 1 | 0.528 | BB | 0.6167 | 1101.03894 | 21.03321 | 0.1902 |
| 2 | 14.119 | BV E | 0.4632 | 164.69075 | 5.59891 | 0.0284 |
| 3 | 15.249 | VB R | 0.5516 | 9671.69141 | 239.24132 | 1.6704 |
| 4 | 17.959 | BV | 0.3664 | 2142.70776 | 90.45295 | 0.3701 |
| 5 | 18.472 | VV | 0.6732 | 5969.29004 | 132.60310 | 1.0309 |
| 6 | 19.578 | VB | 0.3317 | 1066.45483 | 49.43738 | 0.1842 |
| 7 | 20.214 | BB | 0.3095 | 253.06665 | 12.97635 | 0.0437 |
| 8 | 21.551 | BB | 0.4263 | 220.48274 | 8.39689 | 0.0381 |
| 9 | 22.357 | BB | 0.4473 | 96.42830 | 3.52292 | 0.0167 |
| 10 | 23.520 | BB | 0.4871 | 410.94452 | 13.27599 | 0.0710 |
| 11 | 24.955 | BB | 0.4852 | 211.48904 | 6.79345 | 0.0365 |
| 12 | 26.193 | BB | 0.7124 | 3931.22241 | 85.18539 | 0.6789 |
| 13 | 28.525 | BB | 0.6146 | 1306.09924 | 32.79579 | 0.2256 |
| 14 | 30.621 | BB | 1.0198 | 1779.33972 | 24.25771 | 0.3073 |
| 15 | 39.082 | BB | 0.7377 | 561.51282 | 11.66374 | 0.0970 |
| 16 | 41.692 | BB | 0.7937 | 346.32629 | 6.03301 | 0.0598 |
| 17 | 43.237 | BV | 0.5722 | 497.48322 | 13.42971 | 0.0859 |
| 18 | 44.151 | VB | 0.7191 | 1433.59985 | 29.49503 | 0.2476 |
| 19 | 46.570 | BB | 0.7071 | 2861.60132 | 59.92758 | 0.4942 |
| 20 | 49.486 | BV | 0.3958 | 340.23108 | 13.24181 | 0.0588 |
| 21 | 50.143 | VV | 0.4865 | 408.46835 | 12.52484 | 0.0705 |
| 22 | 50.998 | VB | 0.5060 | 396.05148 | 12.03249 | 0.0684 |
| 23 | 52.011 | BB | 0.5605 | 821.35675 | 20.98999 | 0.1419 |
| 24 | 54.044 | BB | 0.4779 | 275.80298 | 8.33897 | 0.0476 |
| 25 | 56.107 | BB | 0.6448 | 287.79498 | 6.23215 | 0.0497 |
| 26 | 57.350 | BB | 0.3854 | 103.93336 | 4.24933 | 0.0179 |
| 27 | 59.663 | BV | 0.5448 | 1848.47217 | 49.59890 | 0.3192 |
| 28 | 61.425 | VV | 0.7881 | 1524.10779 | 26.14486 | 0.2632 |
| 29 | 62.067 | VB | 0.5398 | 654.24908 | 16.81854 | 0.1130 |
| 30 | 64.157 | BV | 0.6156 | 1.04515e4 | 248.04784 | 1.8050 |
| 31 | 65.531 | VV | 0.6368 | 5685.10107 | 121.29472 | 0.9819 |
| 32 | 66.908 | VB | 0.4894 | 5107.24219 | 149.20227 | 0.8821 |
| 33 | 68.813 | BB | 0.5507 | 925.77588 | 25.31443 | 0.1599 |
| 34 | 70.114 | BV | 0.4242 | 1.81176e4 | 644.06683 | 3.1290 |
| 35 | 70.989 | VV | 1.2144 | 1.07813e5 | 1425.38989 | 18.6199 |
| 36 | 75.065 | VV R | 1.8260 | 3.66922e5 | 2354.59595 | 63.3696 |
| 37 | 80.877 | VB E | 0.6215 | 1477.00659 | 32.41837 | 0.2551 |
| 38 | 82.831 | BV | 0.4587 | 2246.90430 | 72.26665 | 0.3881 |
| 39 | 83.548 | VV | 0.4979 | 2598.78394 | 73.96375 | 0.4488 |
| 40 | 84.718 | VB | 0.4614 | 637.73248 | 20.81724 | 0.1101 |
| 41 | 87.498 | BB | 0.5285 | 523.65375 | 13.86796 | 0.0904 |
| 42 | 88.577 | BB | 0.3692 | 104.56291 | 4.56498 | 0.0181 |

| Peak # | RetTime [min] | Type | Width [min] | Area [mAU*s] | Height [mAU] | Area % |
|---|---|---|---|---|---|---|
| 43 | 89.843 | BB | 0.5450 | 1.48505e4 | 396.43347 | 2.5648 |
| 44 | 92.470 | BV | 0.4878 | 687.74866 | 21.81660 | 0.1188 |
| 45 | 93.136 | VB | 0.3707 | 184.36865 | 7.60974 | 0.0318 |

Totals : 5.79019e5 6627.96299

Signal 2: DAD1 B, Sig=254,4 Ref=off

| Peak # | RetTime [min] | Type | Width [min] | Area [mAU*s] | Height [mAU] | Area % |
|---|---|---|---|---|---|---|
| 1 | 17.948 | BV | 0.4078 | 2920.24756 | 108.58186 | 0.8170 |
| 2 | 18.474 | VV | 0.7051 | 6304.00049 | 133.45381 | 1.7638 |
| 3 | 19.473 | VB | 0.3557 | 1527.19006 | 55.73410 | 0.4273 |
| 4 | 20.876 | BB | 0.3445 | 36.83987 | 1.62509 | 0.0103 |
| 5 | 21.608 | BB | 0.4047 | 76.90849 | 3.02420 | 0.0215 |
| 6 | 23.538 | BB | 0.5816 | 174.41568 | 4.37046 | 0.0488 |
| 7 | 26.194 | BB | 0.7944 | 7048.95850 | 134.29701 | 1.9722 |
| 8 | 30.580 | BB | 0.6004 | 178.69949 | 4.32176 | 0.0500 |
| 9 | 31.757 | BB | 0.5931 | 130.60226 | 3.02877 | 0.0365 |
| 10 | 39.084 | BB | 0.7213 | 362.68185 | 7.18059 | 0.1015 |
| 11 | 43.217 | BV | 0.5384 | 363.54828 | 10.43643 | 0.1017 |
| 12 | 44.157 | VB | 0.7097 | 1867.10803 | 39.20132 | 0.5224 |
| 13 | 46.567 | BB | 0.6651 | 1782.47656 | 40.21806 | 0.4987 |
| 14 | 49.488 | BV | 0.3786 | 149.89558 | 6.10135 | 0.0419 |
| 15 | 50.144 | VB | 0.4304 | 152.88326 | 5.33389 | 0.0428 |
| 16 | 51.011 | BB | 0.4170 | 152.96660 | 5.77576 | 0.0428 |
| 17 | 52.001 | BB | 0.4316 | 284.00626 | 10.24046 | 0.0795 |
| 18 | 54.056 | BB | 0.3719 | 71.77959 | 2.99219 | 0.0201 |
| 19 | 56.066 | BB | 0.6394 | 232.43446 | 5.00894 | 0.0650 |
| 20 | 60.973 | BB | 0.6063 | 285.65381 | 7.27247 | 0.0799 |
| 21 | 62.082 | BB | 0.4020 | 106.53735 | 3.90921 | 0.0298 |
| 22 | 64.736 | BB | 0.5413 | 150.62791 | 3.66670 | 0.0421 |
| 23 | 65.545 | BB | 0.3383 | 933.03510 | 43.83886 | 0.2610 |
| 24 | 66.908 | BB | 0.4031 | 2128.44092 | 79.81077 | 0.5955 |
| 25 | 68.836 | BB | 0.6074 | 520.72437 | 11.88052 | 0.1457 |
| 26 | 70.114 | BV | 0.4131 | 6975.47217 | 255.06895 | 1.9516 |
| 27 | 71.003 | VB | 1.1849 | 7.56578e4 | 1034.88428 | 21.1680 |
| 28 | 75.054 | BB | 1.7685 | 2.36487e5 | 1692.14478 | 66.1655 |
| 29 | 80.349 | BV | 0.4624 | 245.89931 | 7.05942 | 0.0688 |
| 30 | 80.868 | VB | 0.4440 | 402.75925 | 13.12654 | 0.1127 |
| 31 | 82.827 | BV | 0.4519 | 1233.17554 | 40.65776 | 0.3450 |
| 32 | 83.551 | VB | 0.4503 | 1006.54041 | 32.58952 | 0.2816 |
| 33 | 84.704 | BB | 0.3707 | 104.28982 | 4.49439 | 0.0292 |
| 34 | 87.498 | BB | 0.5642 | 347.59988 | 8.51623 | 0.0973 |
| 35 | 89.843 | BB | 0.5691 | 6978.82861 | 176.56265 | 1.9526 |
| 36 | 93.139 | BB | 0.3165 | 35.02140 | 1.71291 | 9.798e-3 |

Totals : 3.57417e5 3998.12203

Signal 3: DAD1 C, Sig=280,4 Ref=off

| Peak # | RetTime [min] | Type | Width [min] | Area [mAU*s] | Height [mAU] | Area % |
|---|---|---|---|---|---|---|
| 1 | 16.385 | BB | 0.4637 | 144.80104 | 4.57011 | 0.0456 |
| 2 | 17.943 | BV | 0.4387 | 1209.71411 | 42.16422 | 0.3813 |
| 3 | 18.492 | VB | 0.6741 | 1035.90906 | 25.93075 | 0.3265 |
| 4 | 19.593 | BB | 0.2530 | 232.39690 | 12.85190 | 0.0733 |
| 5 | 23.494 | BB | 0.6193 | 145.04128 | 3.24596 | 0.0457 |
| 6 | 26.240 | BB | 0.7224 | 1375.03223 | 29.46741 | 0.4334 |
| 7 | 39.065 | BB | 0.8073 | 676.72565 | 12.54506 | 0.2133 |
| 8 | 43.233 | BB | 0.5305 | 289.60397 | 8.74255 | 0.0913 |
| 9 | 44.152 | BB | 0.5186 | 204.73022 | 6.11569 | 0.0645 |
| 10 | 46.566 | BB | 0.6649 | 5112.34570 | 114.49414 | 1.6115 |
| 11 | 49.486 | BV | 0.5190 | 710.27454 | 19.77848 | 0.2239 |
| 12 | 51.009 | VV | 0.5413 | 638.62628 | 17.76775 | 0.2013 |
| 13 | 51.999 | VB | 0.5780 | 789.58923 | 19.60158 | 0.2489 |
| 14 | 54.138 | BB | 0.7387 | 189.03618 | 3.29828 | 0.0596 |
| 15 | 55.764 | BB | 0.3637 | 51.45769 | 2.07204 | 0.0162 |
| 16 | 57.777 | BB | 0.8438 | 331.45465 | 5.08968 | 0.1045 |
| 17 | 61.032 | BB | 0.5915 | 610.51324 | 15.30249 | 0.1924 |
| 18 | 64.727 | BV E | 0.6236 | 473.17307 | 9.93612 | 0.1492 |
| 19 | 65.546 | VV R | 0.4990 | 2702.39209 | 77.85239 | 0.8519 |
| 20 | 66.904 | VB | 0.4857 | 1582.39038 | 46.42501 | 0.4988 |
| 21 | 68.780 | BV | 0.6230 | 552.19647 | 12.60615 | 0.1741 |
| 22 | 70.113 | VV | 0.4196 | 9770.36426 | 352.21674 | 3.0799 |
| 23 | 70.982 | VB | 1.1694 | 4.40156e4 | 608.84363 | 13.8748 |
| 24 | 75.056 | BB | 1.7817 | 2.34086e5 | 1669.61694 | 73.7897 |
| 25 | 80.374 | BV | 0.4494 | 313.50406 | 10.12120 | 0.0988 |
| 26 | 80.869 | VB | 0.4742 | 616.57324 | 18.72351 | 0.1944 |
| 27 | 82.819 | BV | 0.5442 | 829.54584 | 21.30870 | 0.2615 |
| 28 | 83.573 | VB | 0.4809 | 1106.26050 | 33.70967 | 0.3487 |
| 29 | 84.734 | BB | 0.3768 | 143.85767 | 6.06241 | 0.0453 |
| 30 | 87.488 | BB | 0.4973 | 231.98529 | 6.74478 | 0.0731 |
| 31 | 89.843 | BB | 0.5692 | 7030.51367 | 177.83578 | 2.2162 |
| 32 | 93.129 | BB | 0.3150 | 32.30610 | 1.59060 | 0.0102 |

Totals : 3.17234e5 3396.63173

Signal 4: DAD1 D, Sig=365,4 Ref=off

| Peak # | RetTime [min] | Type | Width [min] | Area [mAU*s] | Height [mAU] | Area % |
|---|---|---|---|---|---|---|
| 1 | 51.998 | BB | 0.4451 | 83.50582 | 2.72812 | 0.0290 |
| 2 | 64.776 | BB | 0.5254 | 81.75130 | 2.02312 | 0.0284 |
| 3 | 65.544 | BB | 0.3987 | 379.19598 | 14.24052 | 0.1319 |
| 4 | 66.912 | BB | 0.4517 | 2173.64355 | 70.10752 | 0.7561 |
| 5 | 68.735 | BB | 0.5532 | 124.90044 | 2.89556 | 0.0434 |
| 6 | 70.114 | BV E | 0.4059 | 3075.13477 | 115.03120 | 1.0697 |
| 7 | 71.006 | VV R | 1.1982 | 7.25380e4 | 979.27734 | 25.2329 |
| 8 | 75.053 | VB | 1.7854 | 1.99034e5 | 1414.71216 | 69.2354 |
| 9 | 80.884 | BB | 0.4339 | 376.21335 | 12.54235 | 0.1309 |

| Peak # | RetTime [min] | Type | Width [min] | Area [mAU*s] | Height [mAU] | Area % |
|---|---|---|---|---|---|---|
| 10 | 82.833 | BV | 0.4418 | 1154.96313 | 38.96262 | 0.4018 |
| 11 | 83.550 | VB | 0.4447 | 1346.00061 | 44.02751 | 0.4682 |
| 12 | 84.732 | BB | 0.3808 | 102.89565 | 4.27546 | 0.0358 |
| 13 | 87.498 | BB | 0.4803 | 373.48837 | 11.45886 | 0.1299 |
| 14 | 89.843 | BB | 0.5652 | 6167.32910 | 156.70401 | 2.1453 |
| 15 | 92.431 | BB | 0.5146 | 463.24481 | 13.29106 | 0.1611 |

Totals : 2.87474e5 2882.27741

*** End of Report ***
