# Supplementary material for: The In Vitro Antioxidant and Anti-Inflammatory Activities of Selected Australian Seagrasses
Source: Life (Basel). 2024 May 30;14(6):710. doi: 10.3390/life14060710 (PMC11205046; doi:10.3390/life14060710)
Supplement: Supplementary file 1 [file life-14-00710-s001.zip › Supporting Information_Revised.pdf]

The In Vitro Antioxidant and Anti-Inflammatory Activities of Selected Australian Seagrasses

Matthew J. Perry [1,2,*,†], Mara Curic [1,2,3,†], Abigail L. Scott [4], Edita Ritmejerytė [1], Dyah U. C. Rahayu [5], Paul A. Keller [5], Michael Oelgemöller [3], Karma Yeshi [1,2,‡] and Phurpa Wangchuk [1,2,*,‡]

[1] College of Public Health, Medical and Veterinary Sciences, James Cook University, Cairns, QLD 4878, Australia; karma.yeshi@my.jcu.edu.au (K.Y.)
[2] Australian Institute of Tropical Health and Medicine, James Cook University, Cairns, QLD 4878, Australia
[3] Hochschule Fresenius, Faculty of Chemistry & Biology, University of Applied Sciences, Limburger Strasse 2, 65510 Idstein, Germany
[4] Centre of Tropical Water & Aquatic Ecosystem Research, James Cook University, Cairns, QLD 4878, Australia; abbi.scott1@jcu.edu.au
[5] School of Chemistry and Molecular Bioscience, Molecular Horizons, University of Wollongong, Wollongong, NSW 2522, Australia
* Correspondence: matthew.perry1@jcu.edu.au (M.J.P.); phurpa.wangchuk@jcu.edu.au (P.W.)
† Equally contributed as first authors.
‡ Equally contributed as senior authors.

Table of Contents

Figure S1: The ^{1}H NMR spectrum of 4-hydroxybenzoic acid **1** (500 MHz, CD_3OD, 298 K). . 2

Figure S2: The ^{13}C NMR spectrum of 4-hydroxybenzoic acid **1** (126 MHz, CD_3OD, 298 K). 2

Figure S3: The ^{1}H NMR spectrum of luteolin **2** (500 MHz, CD_3OD, 298 K)........................3

Figure S4: The ^{13}C NMR spectrum of luteolin **2** (126 MHz, CD_3OD, 298 K). 3

Figure S5: The 2D COSY spectrum of luteolin **2** (CD_3OD, 298 K)..4

Figure S6: The 2D HMBC spectrum of luteolin **2** (CD_3OD, 298 K). 4

Figure S7: The 2D HSQC spectrum of luteolin **2** (CD_3OD, 298 K)..5

Figure S8: The 2D NOESY spectrum of luteolin **2** (CD_3OD, 298 K).....................................5

Figure S9: The ^{1}H NMR spectrum of apigenin **3** (500 MHz, CD_3OD, 298 K). 6

Figure S10: The ^{13}C NMR spectrum of apigenin **3** (126 MHz, CD_3OD, 298 K).....................6

Figure S11: The 2D COSY spectrum of apigenin **3** (CD_3OD, 298 K). 7

Figure S12: The 2D HMBC spectrum of apigenin **3** (CD_3OD, 298 K)...................................7

Figure S13: The 2D HSQC spectrum of apigenin **3** (CD_3OD, 298 K). 8

Figure S14: The 2D NOESY spectrum of apigenin **3** (CD_3OD, 298 K). 8

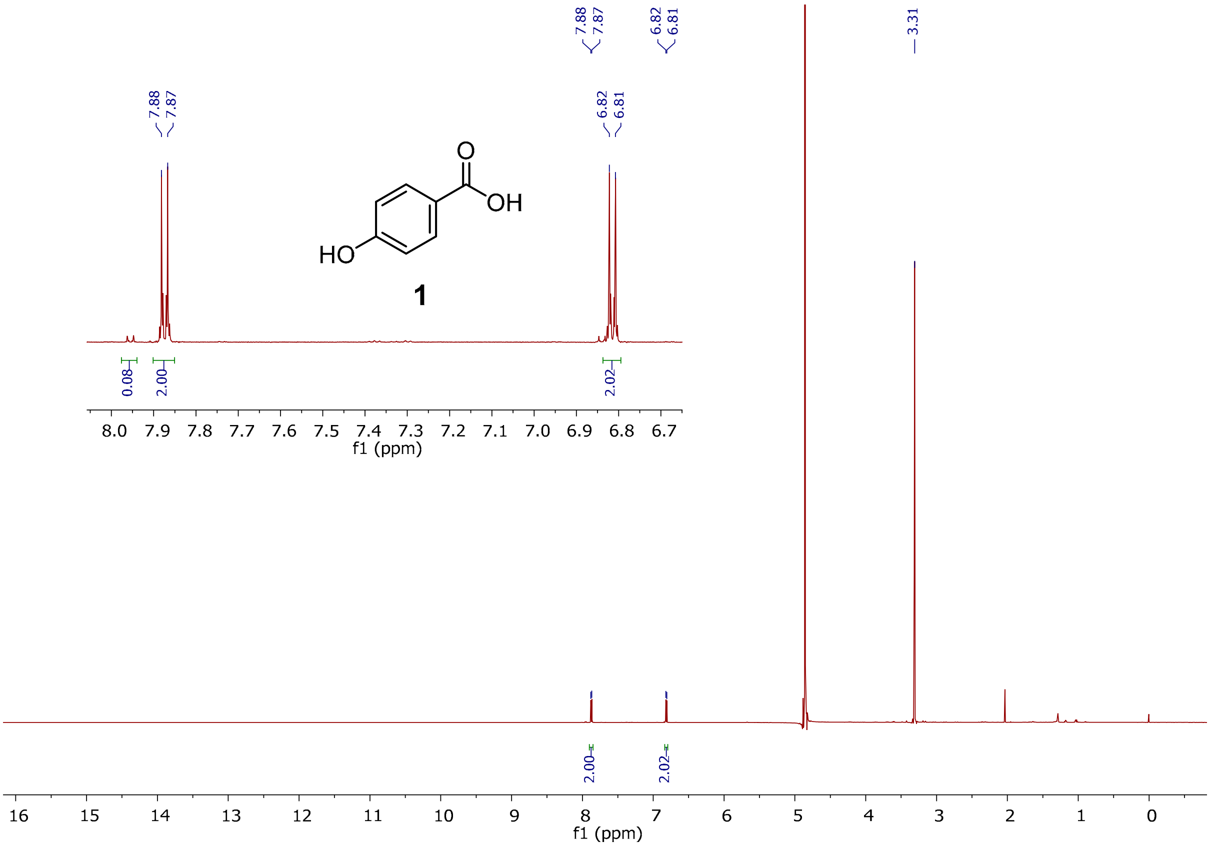

Figure S1: The ^{1}H NMR spectrum of 4-hydroxybenzoic acid **1** (500 MHz, CD_3OD, 298 K).

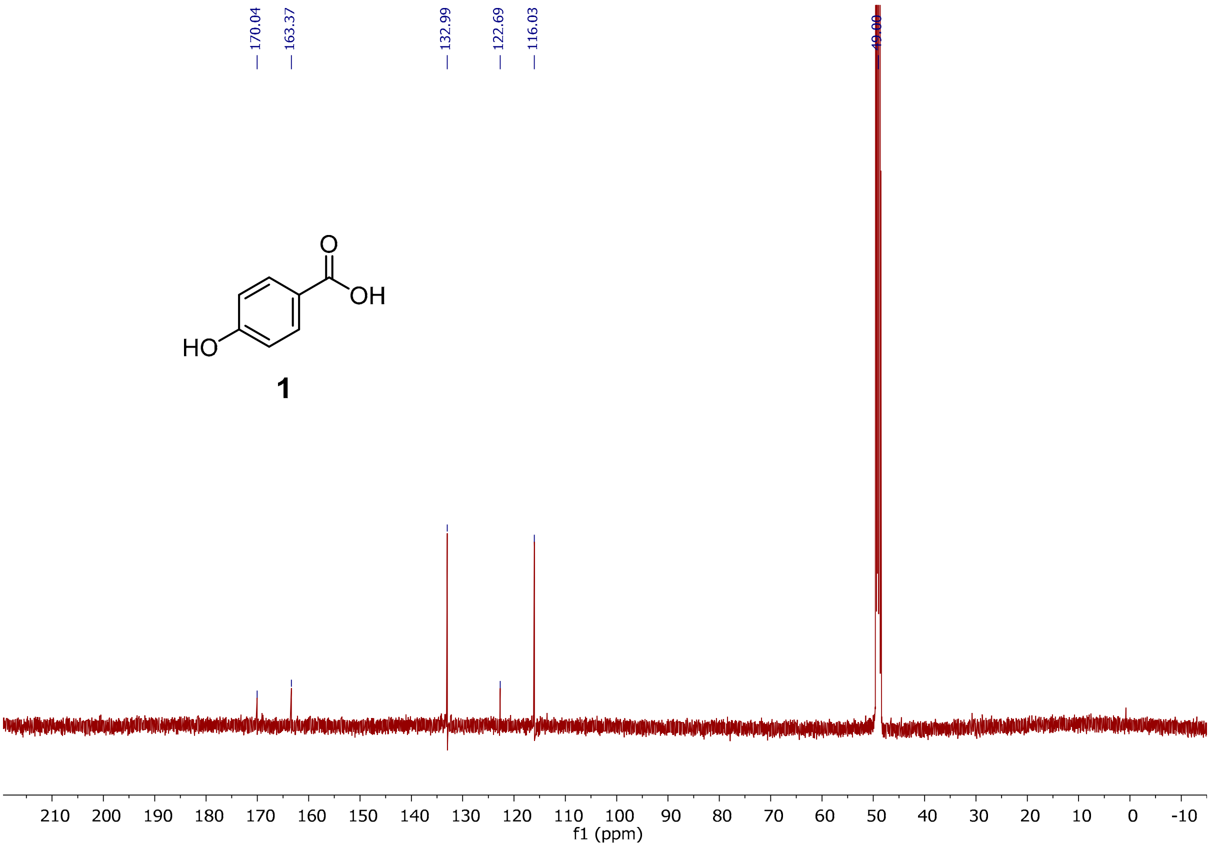

Figure S2: The ^{13}C NMR spectrum of 4-hydroxybenzoic acid **1** (126 MHz, CD_3OD, 298 K).

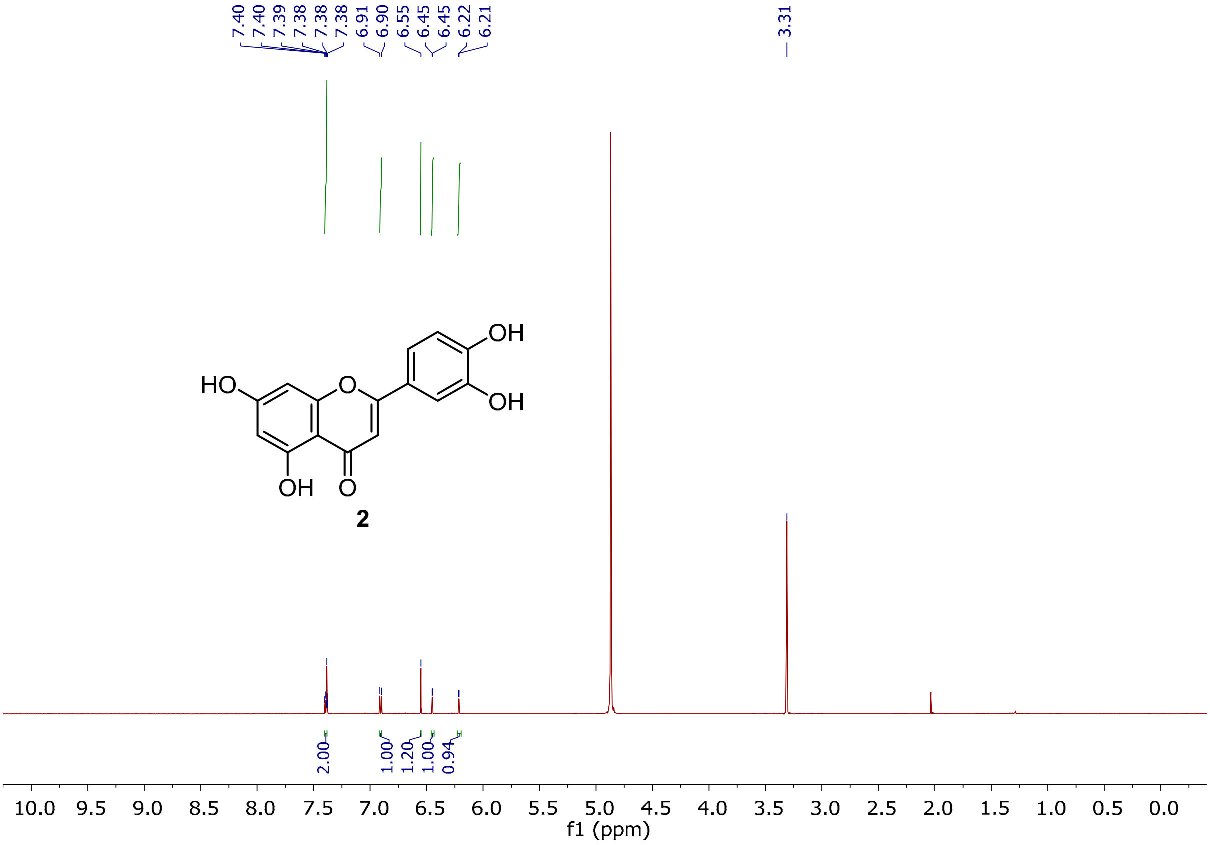

Figure S3: The ^{1}H NMR spectrum of luteolin **2** (500 MHz, CD_3OD, 298 K).

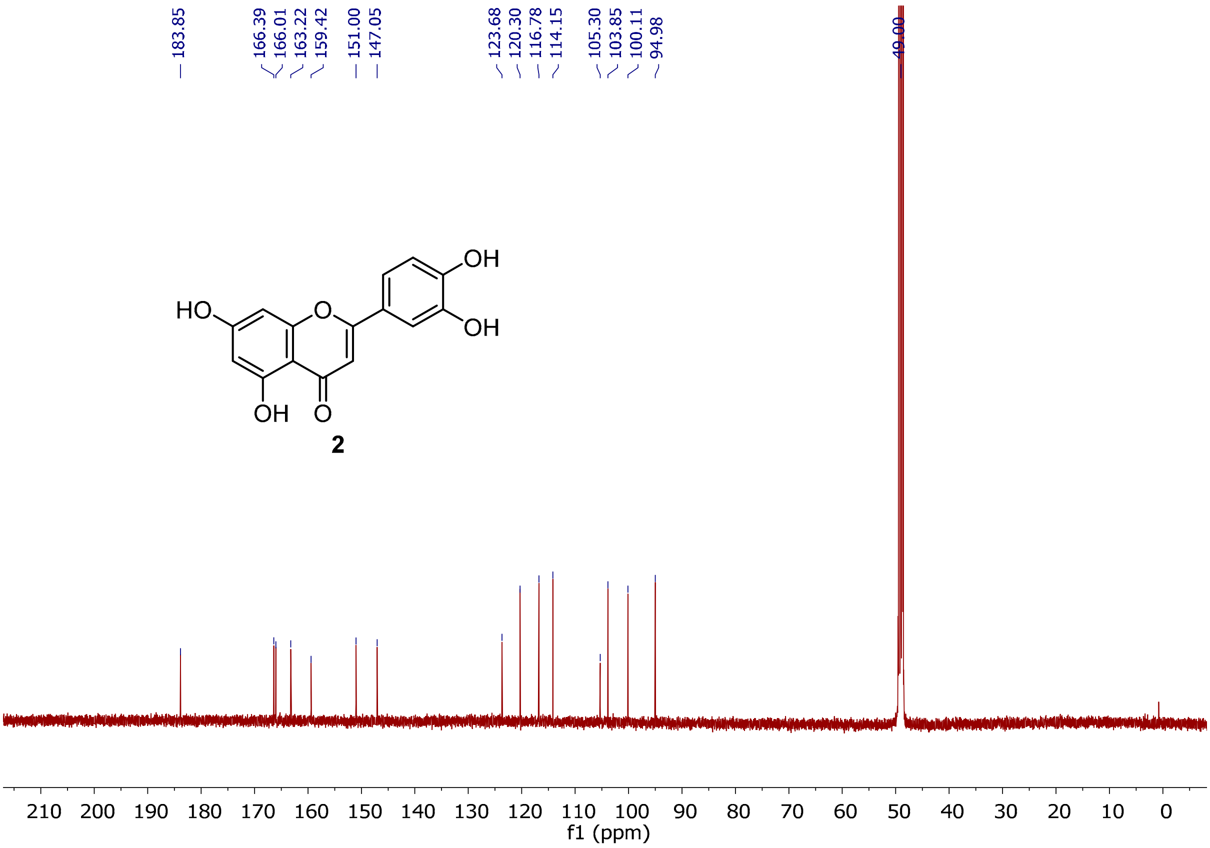

Figure S4: The ^{13}C NMR spectrum of luteolin **2** (126 MHz, CD_3OD, 298 K).

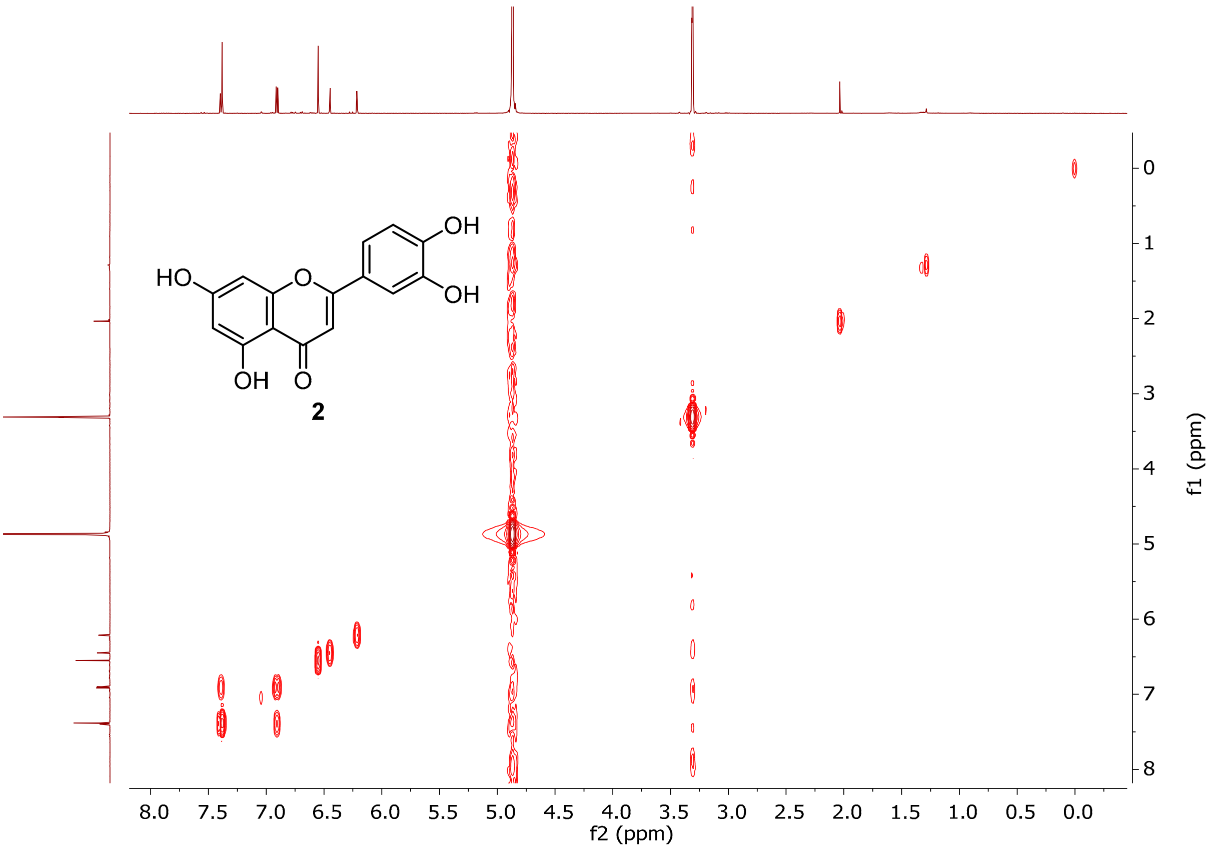

Figure S5: The 2D COSY spectrum of luteolin **2** (CD_3OD, 298 K).

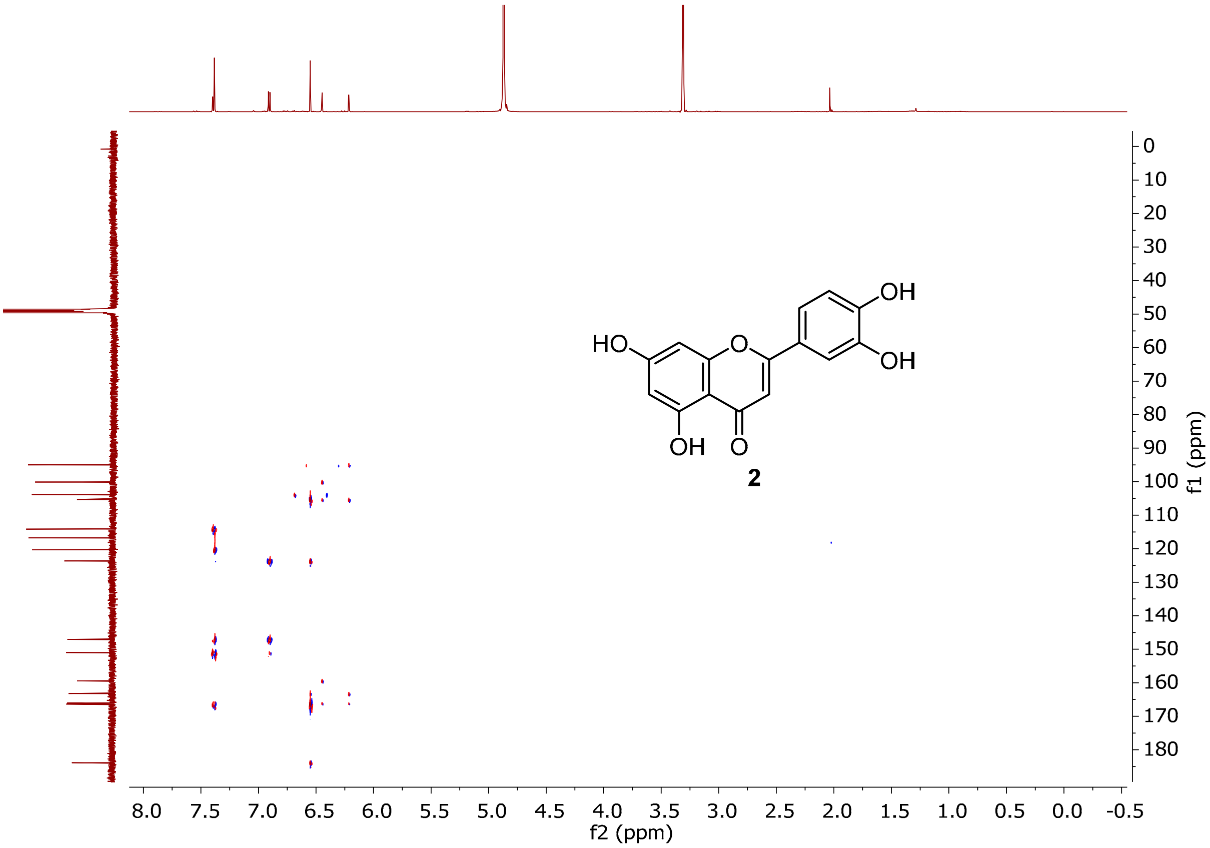

Figure S6: The 2D HMBC spectrum of luteolin **2** (CD_3OD, 298 K).

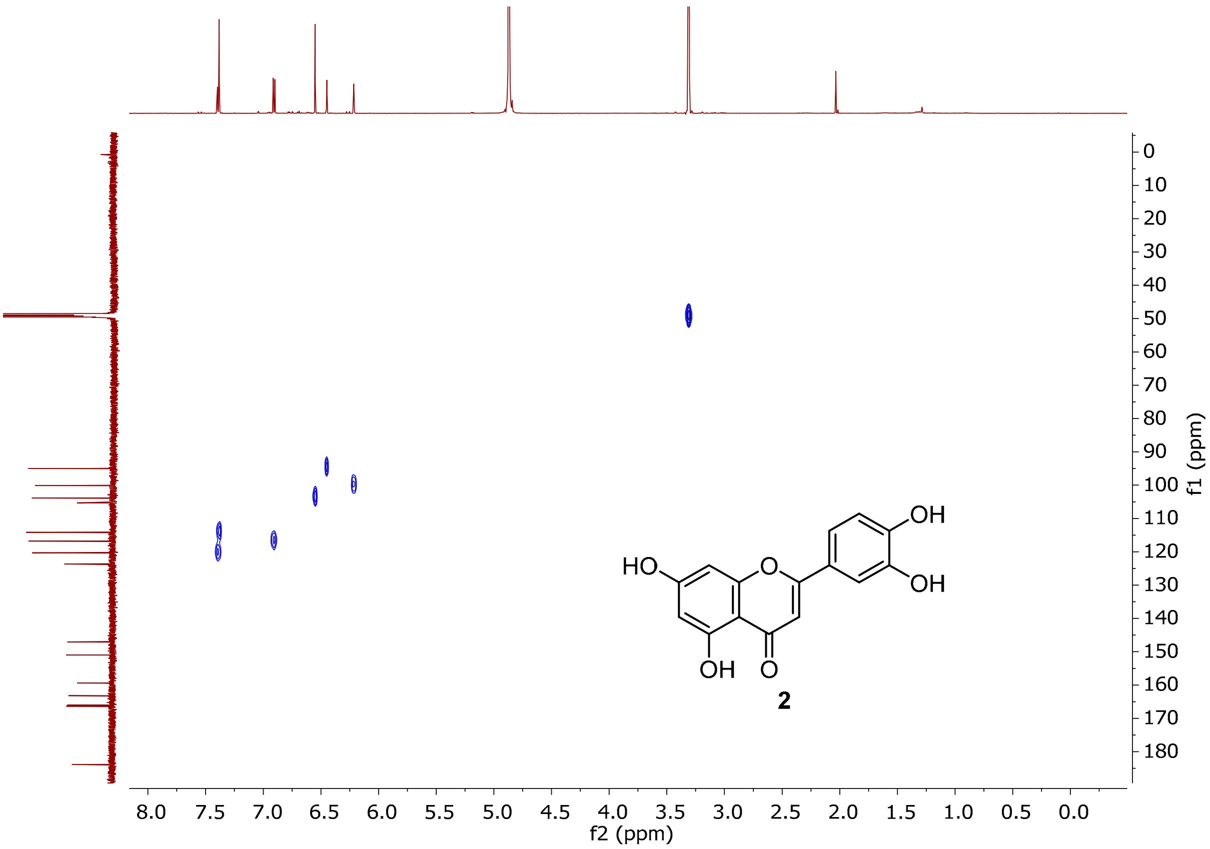

Figure S7: The 2D HSQC spectrum of luteolin **2** (CD_3OD, 298 K).

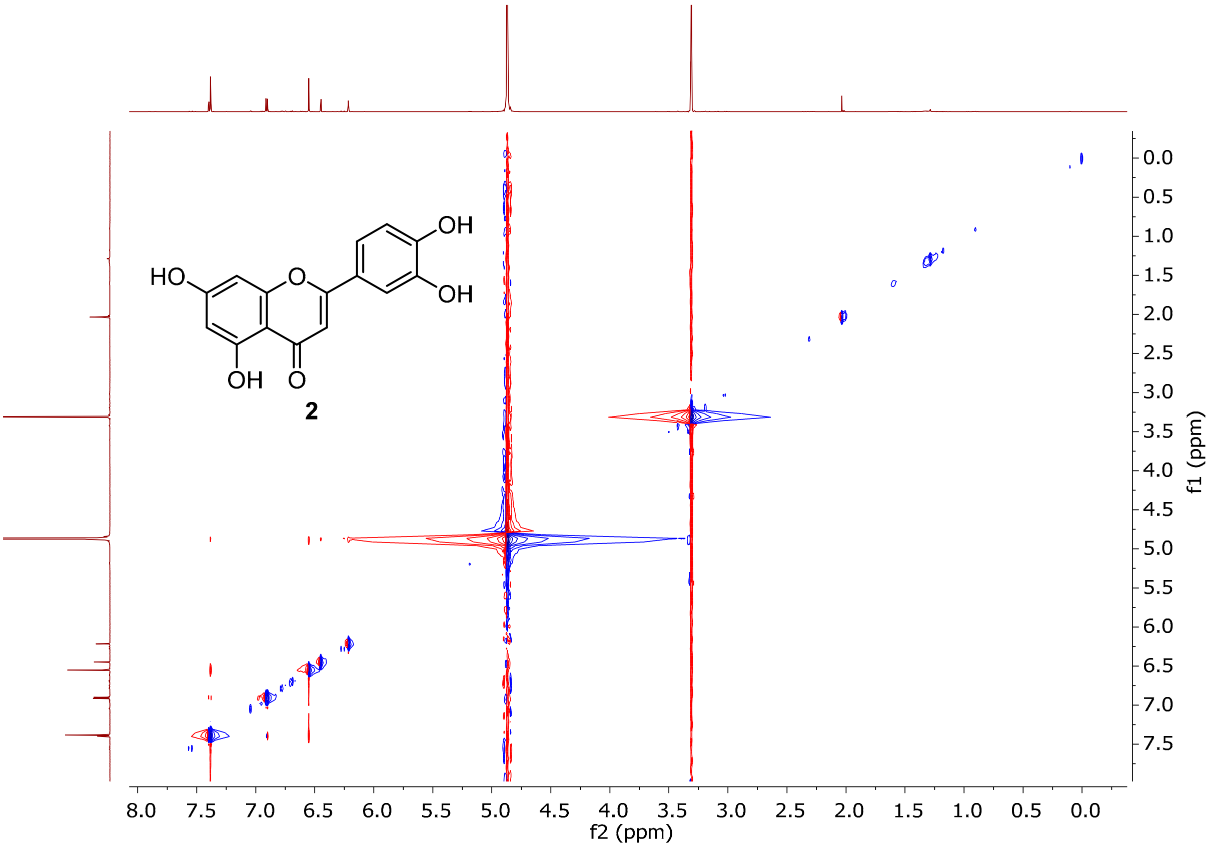

Figure S8: The 2D NOESY spectrum of luteolin **2** (CD_3OD, 298 K).

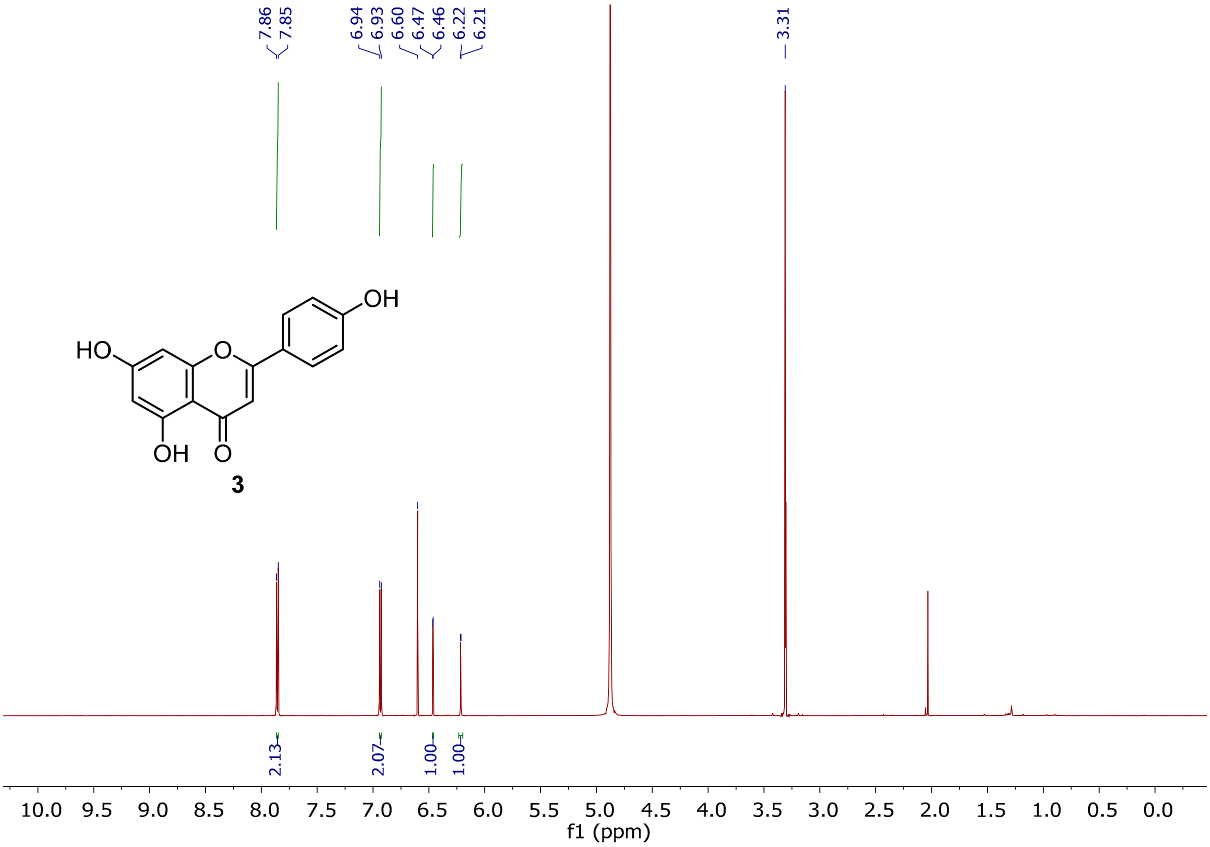

Figure S9: The ^{1}H NMR spectrum of apigenin **3** (500 MHz, CD_3OD, 298 K).

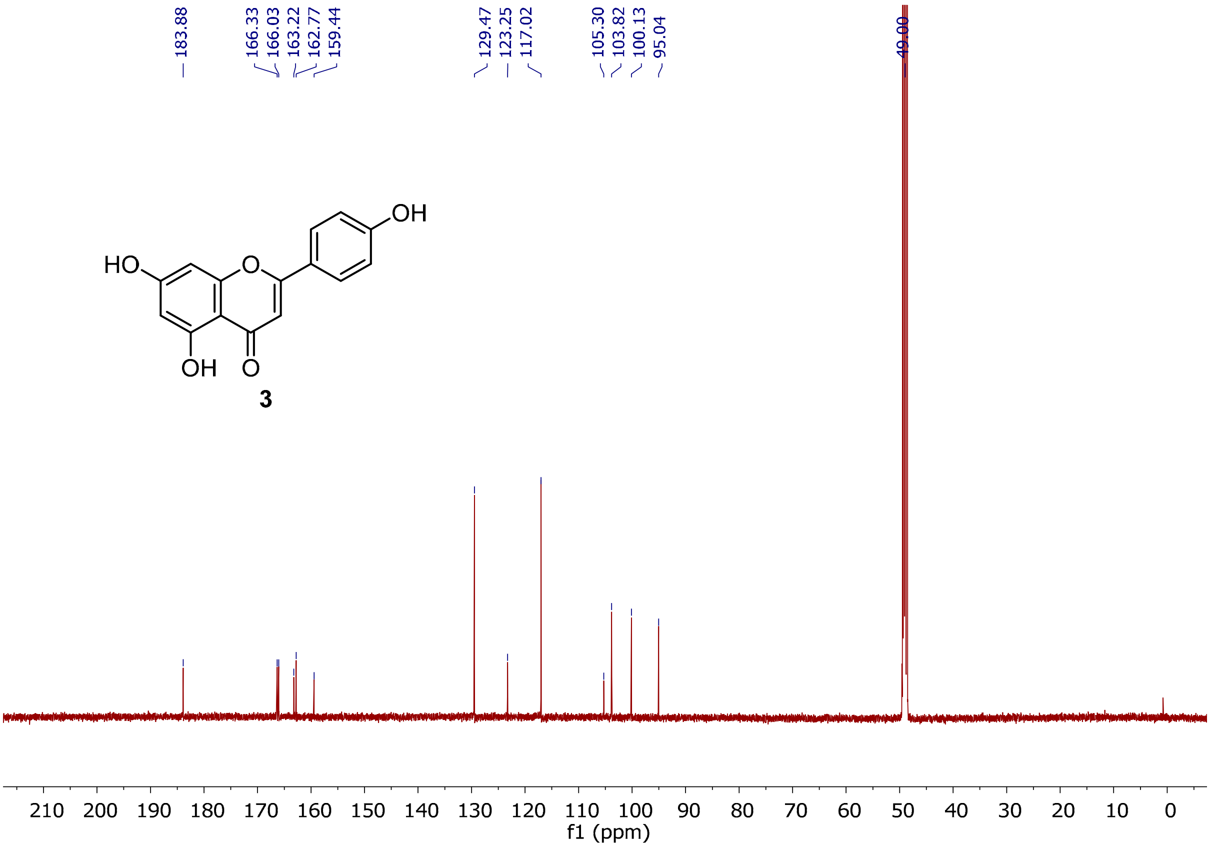

Figure S10: The ^{13}C NMR spectrum of apigenin **3** (126 MHz, CD_3OD, 298 K).

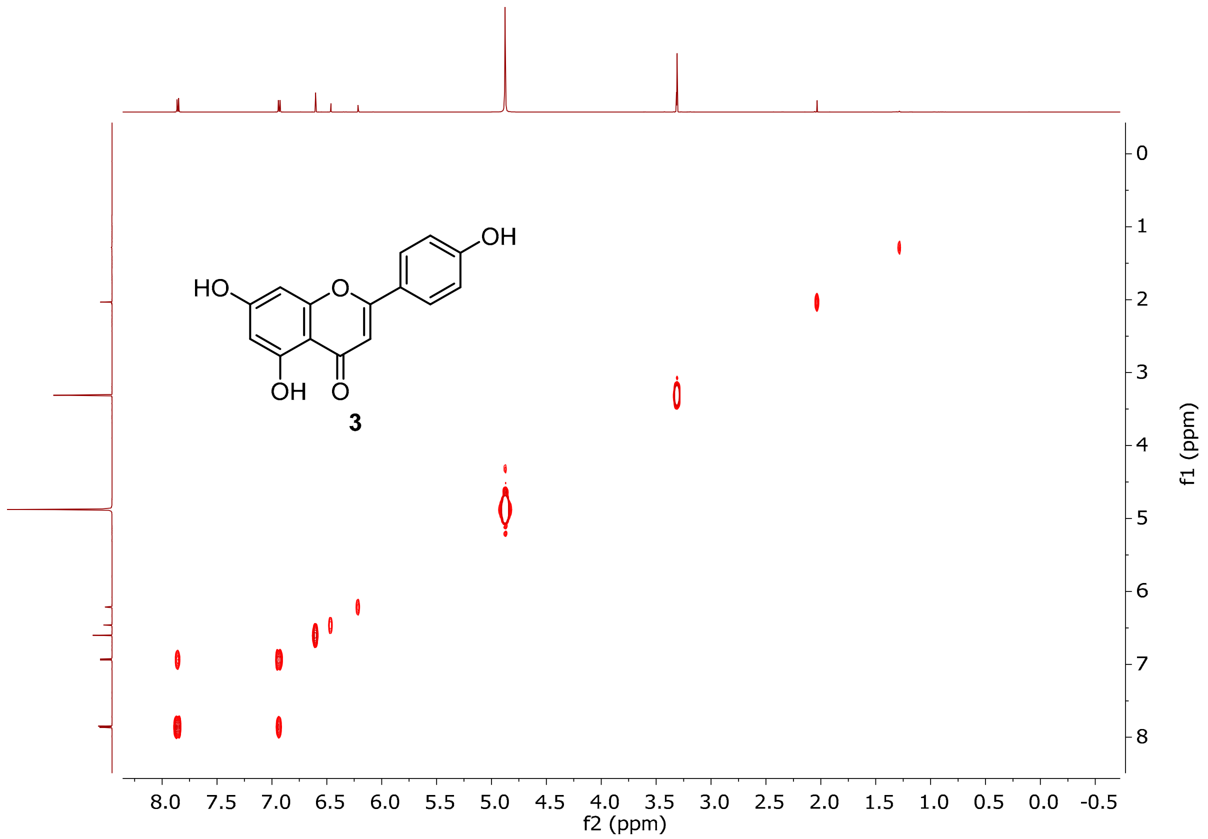

Figure S11: The 2D COSY spectrum of apigenin **3** (CD_3OD, 298 K).

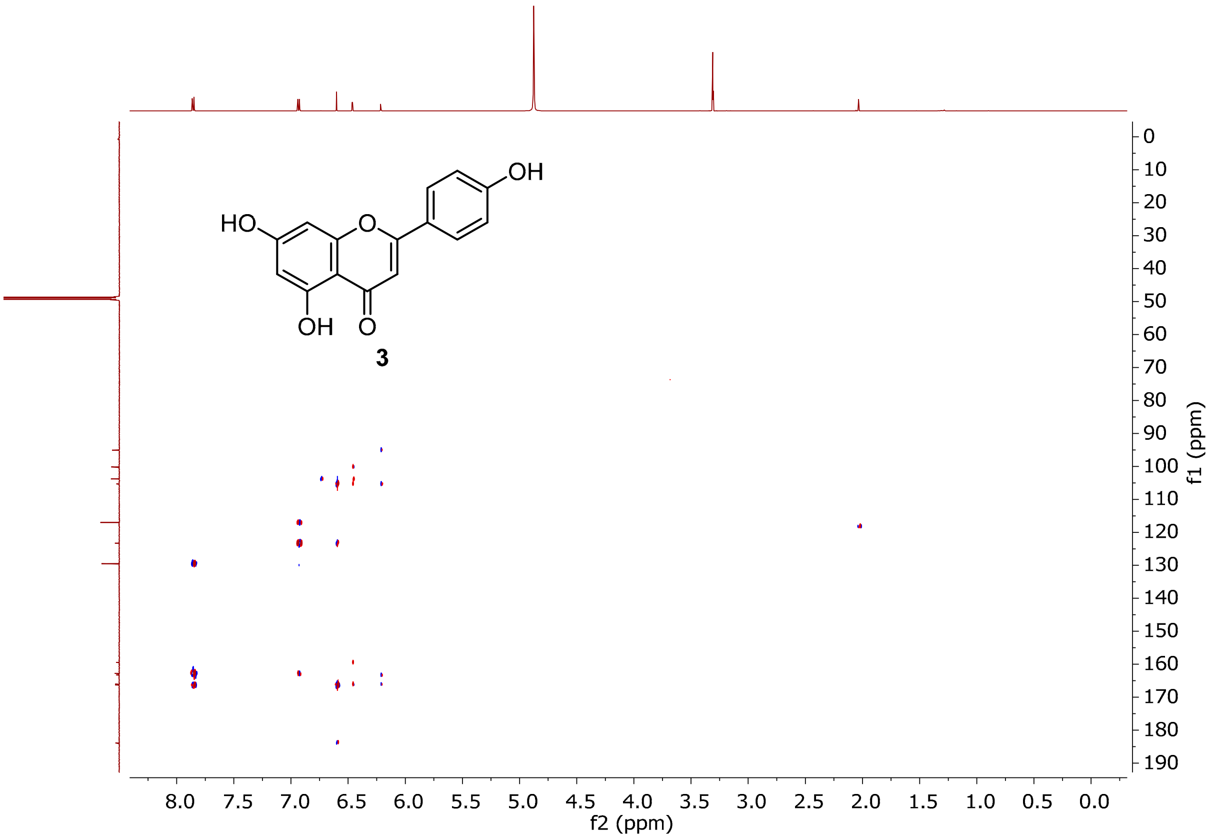

Figure S12: The 2D HMBC spectrum of apigenin **3** (CD_3OD, 298 K).

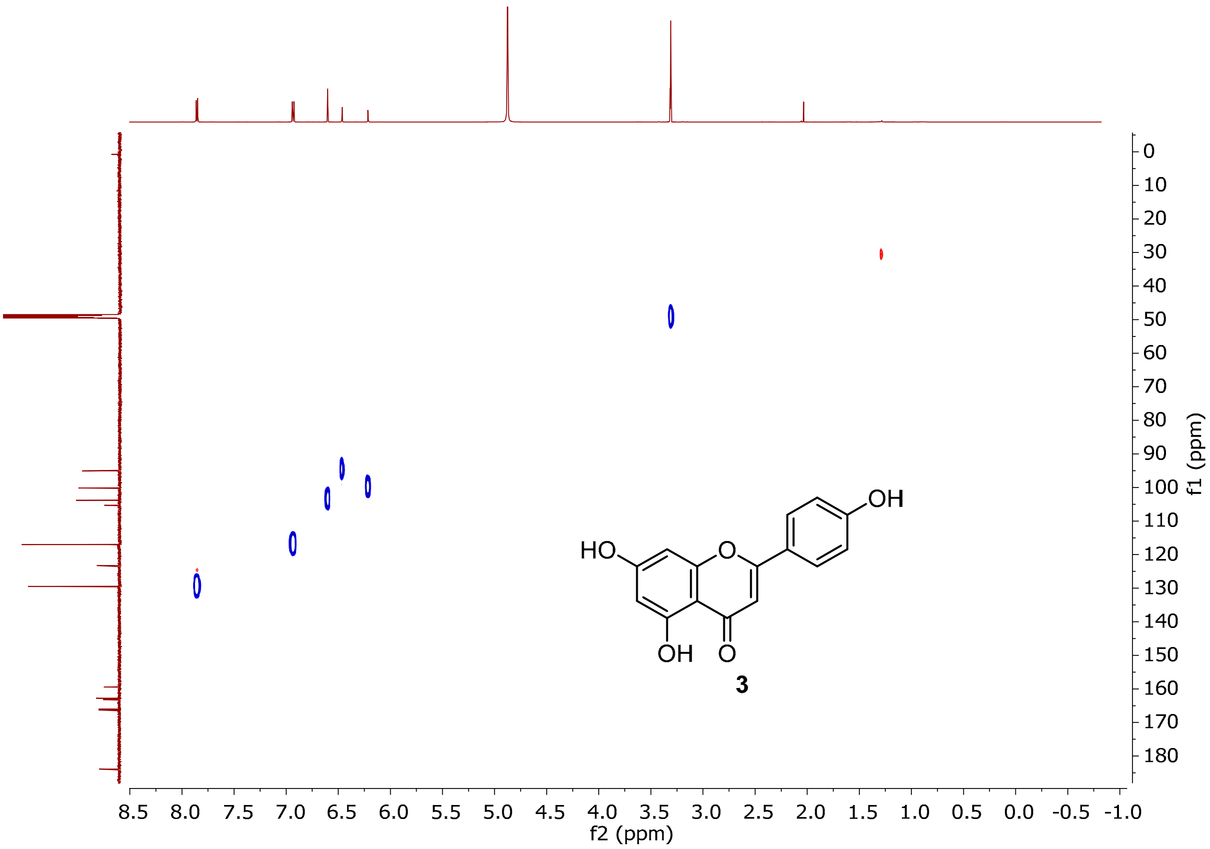

Figure S13: The 2D HSQC spectrum of apigenin **3** (CD_3OD, 298 K).

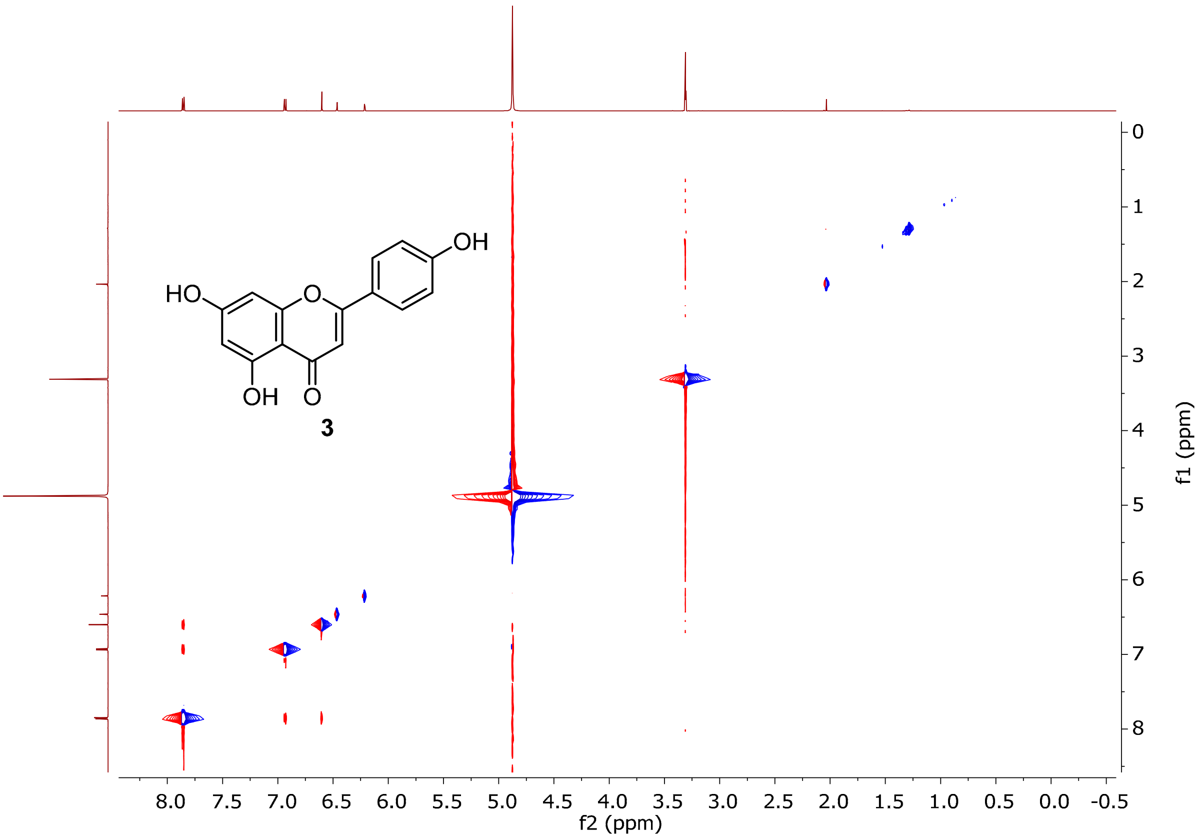

Figure S14: The 2D NOESY spectrum of apigenin **3** (CD_3OD, 298 K).
